# Author Correction: The genetic prehistory of the Baltic Sea region

**DOI:** 10.1038/s41467-018-03872-y

**Published:** 2018-04-11

**Authors:** Alissa Mittnik, Chuan-Chao Wang, Saskia Pfrengle, Mantas Daubaras, Gunita Zariņa, Fredrik Hallgren, Raili Allmäe, Valery Khartanovich, Vyacheslav Moiseyev, Mari Tõrv, Anja Furtwängler, Aida Andrades Valtueña, Michal Feldman, Christos Economou, Markku Oinonen, Andrejs Vasks, Elena Balanovska, David Reich, Rimantas Jankauskas, Wolfgang Haak, Stephan Schiffels, Johannes Krause

**Affiliations:** 1 Department of Archaeogenetics, Max Planck Institute for the Science of Human History, 07745 Jena, Germany; 2 Institute for Archaeological Sciences, Archaeo- and Palaeogenetics, University of Tübingen, 72070 Tübingen, Germany; 3 Department of Anthropology and Ethnology, Xiamen University, 361005 Xiamen, China; 4 Department of Archaeology, Lithuanian Institute of History, 01108 Vilnius, Lithuania; 5 Institute of Latvian History, University of Latvia, Riga, LV-1050 Latvia; 6 The Cultural Heritage Foundation, 72212 Västerås, Sweden; 7 Archaeological Research Collection, Tallinn University, 10130 Tallinn, Estonia; 8 Peter the Great Museum of Anthropology and Ethnography (Kunstkamera) RAS, 199034 St. Petersburg, Russia; 9 Institute of History and Archaeology, University of Tartu, 50090 Tartu, Estonia; 10 Archaeological Research Laboratory, Stockholm University, 11418 Stockholm, Sweden; 11 Finnish Museum of Natural History - LUOMUS, University of Helsinki, 00014 Helsinki, Finland; 12 Research Centre for Medical Genetics, 115478 Moscow, Russia; 13 Department of Genetics, Harvard Medical School, Boston, MA 02115 USA; 14 Broad Institute of Harvard and MIT, Cambridge, MA 02142 USA; 15 Howard Hughes Medical Institute, Harvard Medical School, Boston, MA 02115 USA; 16 Department of Anatomy, Histology and Anthropology, Vilnius University, 03101 Vilnius, Lithuania; 17 School of Biological Sciences, The University of Adelaide, Adelaide, SA 5005 Australia

Correction to: *Nature Communications* 10.1038/s41467-018-02825-9, published online: 30 January 2018

The original version of this Article omitted references to previous work in ‘Piličiauskas, G. & Heron, C. Aquatic radiocarbon reservoir offsets in the southeastern Baltic. Radiocarbon 57, 539–556 (2015)’ and ‘Piličiauskas, G. et al. The transition from foraging to farming (7000–500 cal BC) in the SE Baltic: A re-evaluation of chronological and palaeodietary evidence from human remains. Journal of Archaeological Science: Reports 14, 530–542 (2017) ’. These have been added as References 28 and 30 in a new sentence added to the end of the legend of Table 1: ‘Radiocarbon dates for Spiginas1 and Donkalnis7 were first reported in ref. 28, radiocarbon dates for Spiginas2, Donkalnis6, Kretuonas5, Gyvakarai, and Turlojiškė1 were first reported in ref. 30.’

The first sentence of the third paragraph of the ‘Dynamic forager networks in the Eastern Baltic Neolithic’ section of the Results originally read ‘One Narva individual, Spiginas1, dated to ca. 4440–4240 calBCE, belongs to a mitochondrial haplogroup of the H branch, normally associated with the Neolithic expansion into Europe, but shows no evidence of Neolithic farmer ancestry on the nuclear level suggesting that this haplogroup might have been present already in foraging groups (Table 1, Supplementary Table 5).’ The correct version adds ‘(ref. 28)’ after ‘Spiginas1’.

The last sentence of the second paragraph of the ‘New networks of contact during the LNBA’ section of the Results originally read ‘The individual Spiginas2, dated to a very late period of the LN (2130–1750 calBCE), stands out in that it shares an excess of alleles with European forager groups when compared to the Yamnaya populations, with the top hits being Switzerland_HG, WHG, Baltic Mesolithic and Baltic EMN Narva (Supplementary Table 7).’ The correct version adds ‘(ref. 30)’ after ‘Spiginas2’.Also, the last sentence of the second paragraph of the Discussion incorrectly read ‘This scenario is also supported by the finding that three Mesolithic hunter-gatherers excavated at the coast of Norway carry a higher proportion of EHG ancestry compared to the individuals from inland Sweden.’ The correct version adds ‘37’ after ‘Sweden’.

This has been corrected in both the PDF and HTML versions of the Article.

The original version of the Supplementary Information associated with this Article also omitted these two references. They have been added as Supplementary References 171 and  172, respectively.

The last sentence of the ‘Biržai, Lithuania’ section of Supplementary Note 2 originally read ‘The grave was partially destroyed during the construction works in 2013 and then rescue excavations took place in 2014 uncovering the single grave dating to the Late Neolithic period^60,^.’ The correct version adds^171^ to the end.

The seventh to ninth sentences of the ‘Donkalnis settlement and burial site’ section of Supplementary Note 2 originally read ‘Three out of 7 graves (no. 2-4) were C14 dated to the Middle Mesolithic, Early Neolithic and Late Mesolithic periods, respectively, but the director of the excavations argues that 3 above-mentioned graves together with one more grave (no. 5) belong to the Middle – Late Mesolithic with the rest of them (no. 1, 6-7) dating to the Late Neolithic period (Butrimas 2012). The authors of this article suggest that there are some flaws considering the chronology of Donkalnis graves. These issues are addressed in more detail further in the text.’ The correct version of this paragraph was changed to read ‘Graves 4 and 5^8,171^ were radiocarbon dated to the Mesolithic, while grave 6 and 7 were directly dated to the Middle Neolithic^171,172^, and grave 1 assigned to the same period based on associated artefacts.’.

The last sentence of the ‘Gyvakarai burial site’ section of Supplementary Note 2 originally read ‘The same year rescue excavations were conducted in the surrounding area of the highly disturbed grave resulting in discovery of a single grave C14 dated to the Late Neolithic^64^ The correct version adds^171^ to the end.

The second to last sentence of the ‘Kretuonas settlement and burial site’ section of Supplementary Note 2 originally read ‘In 1980, six undisturbed graves dating to the Early – Middle Neolithic periods were uncovered in the low-lying and intermittently flooded periphery of dwelling area.’ The correct version adds^8,171^ to the end.

The last sentence of the ‘Spiginas settlement and burial site’ section of Supplementary Note 2 originally read ‘During these excavations 4 graves were discovered and C14 dated to the Middle Mesolithic, Middle Neolithic and Late Neolithic periods^61^.’ The correct version cites^8,62,171,172^ at the end.

The last sentence of the ‘Turlojiškė archaeological complex, Lithuania’ section of Supplementary Note 2 originally read ‘During the excavations 6 additional male graves were discovered and some of them were radiocarbon dated to Bronze age^69^.’ The correct version cites^8,68,171^.

Further, reference 8 of the Supplementary Information file was incorrectly given as ‘*8. Antanaitis-*Jacobs, I. & Girininkas, A. *2002 Periodization and chronology* of the Neolithic in Lithuania. *Archaeologia Baltica* 5, 9–39.’ The correct version changes the reference to *‘8. Antanaitis-*Jacobs, I., Richards, M., Daugnora, L., Jankauskas, R., Ogrinc, N. 2009. Diet in early Lithuanian prehistory and the new stable isotope evidence. *Archaeologia Baltica* 12, 12-30.’

The first sentence of the ‘Popovo, Archangelsk, Russia’ section of Supplementary Note 2 originally incorrectly read ‘The Mesolithic site is located on the bank of the Kinema River, in the Archangelsk region (64°32′N 40°32′E).’ The correct version states ‘61°15′N 38°54′E’ instead of ‘64°32′N 40°32′E’

The HTML has been updated to include a corrected version of the Supplementary Information.

