# Supplementary material for: The genetic prehistory of the Baltic Sea region
Source: Nat Commun. 2018 Jan 30;9:442. doi: 10.1038/s41467-018-02825-9 (PMC5789860; doi:10.1038/s41467-018-02825-9)
Supplement: Supplementary file 1 — Supplementary Information [file 41467_2018_2825_MOESM1_ESM.pdf]

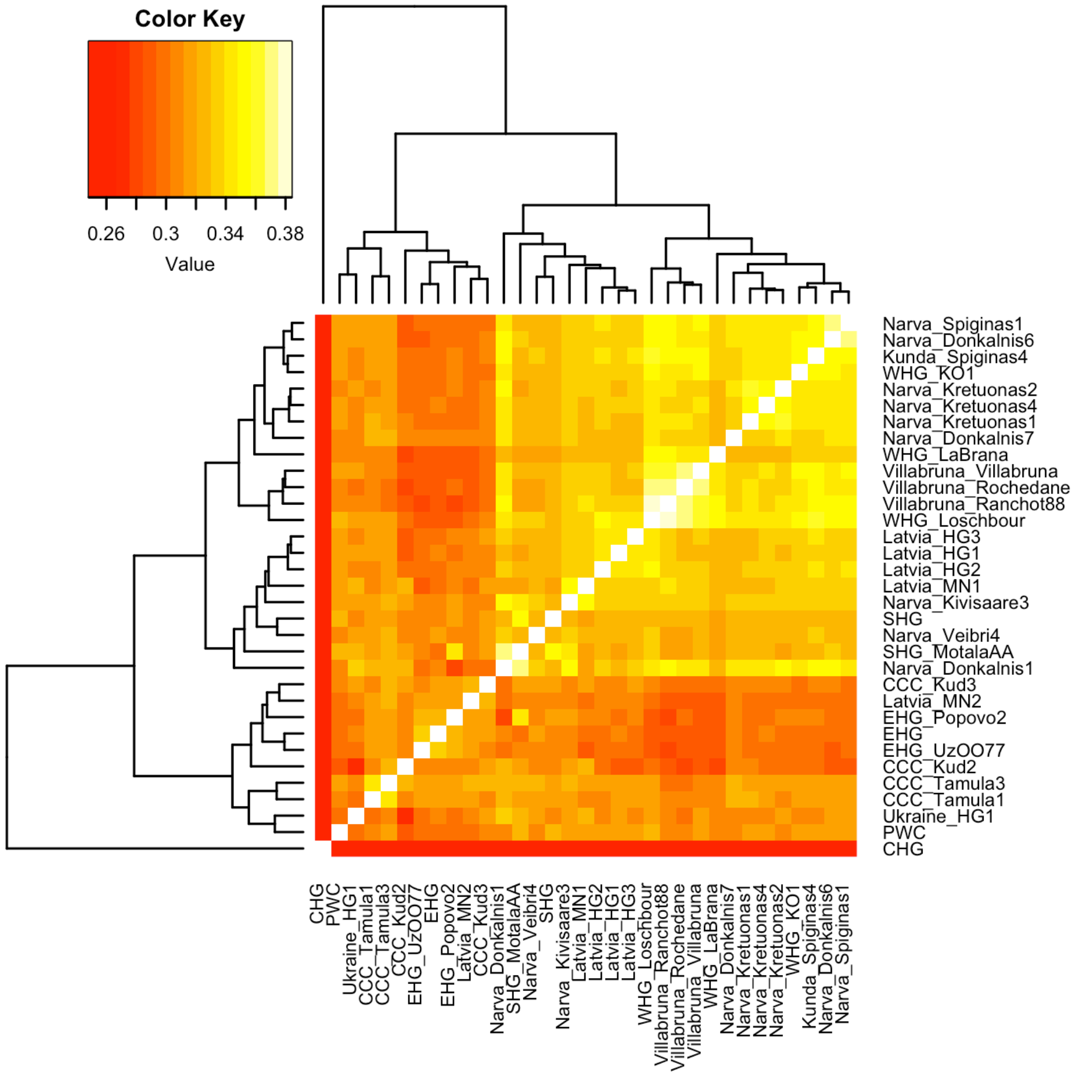

Supplementary Figure 1: Pairwise f3-statistics of late Western Eurasian hunter-gatherers. Eastern Baltic hunter-gatherers fall within the 'Villabruna' cluster (including WHG) of European hunter-gatherers dating after ca. 14,000 BP.

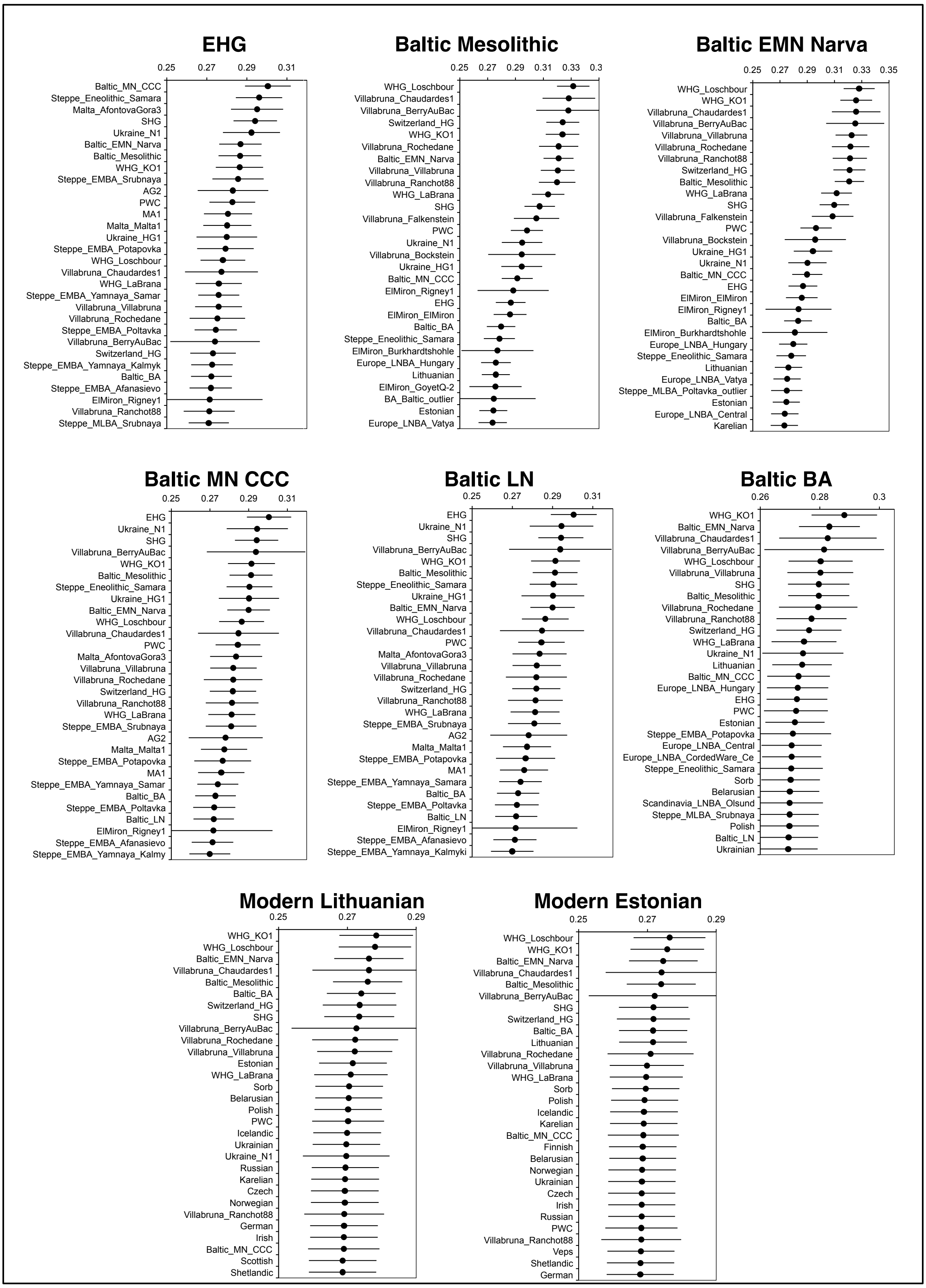

Supplementary Figure 2: Outgroup f_3-statistics for ancient and modern Eastern Baltic populations. Values shown for the statistic f_3(Eastern Baltic Population, *X*; Mbuti), where *X* is a modern or ancient population. The thirty highest hits are shown and error bars represent three standard errors.

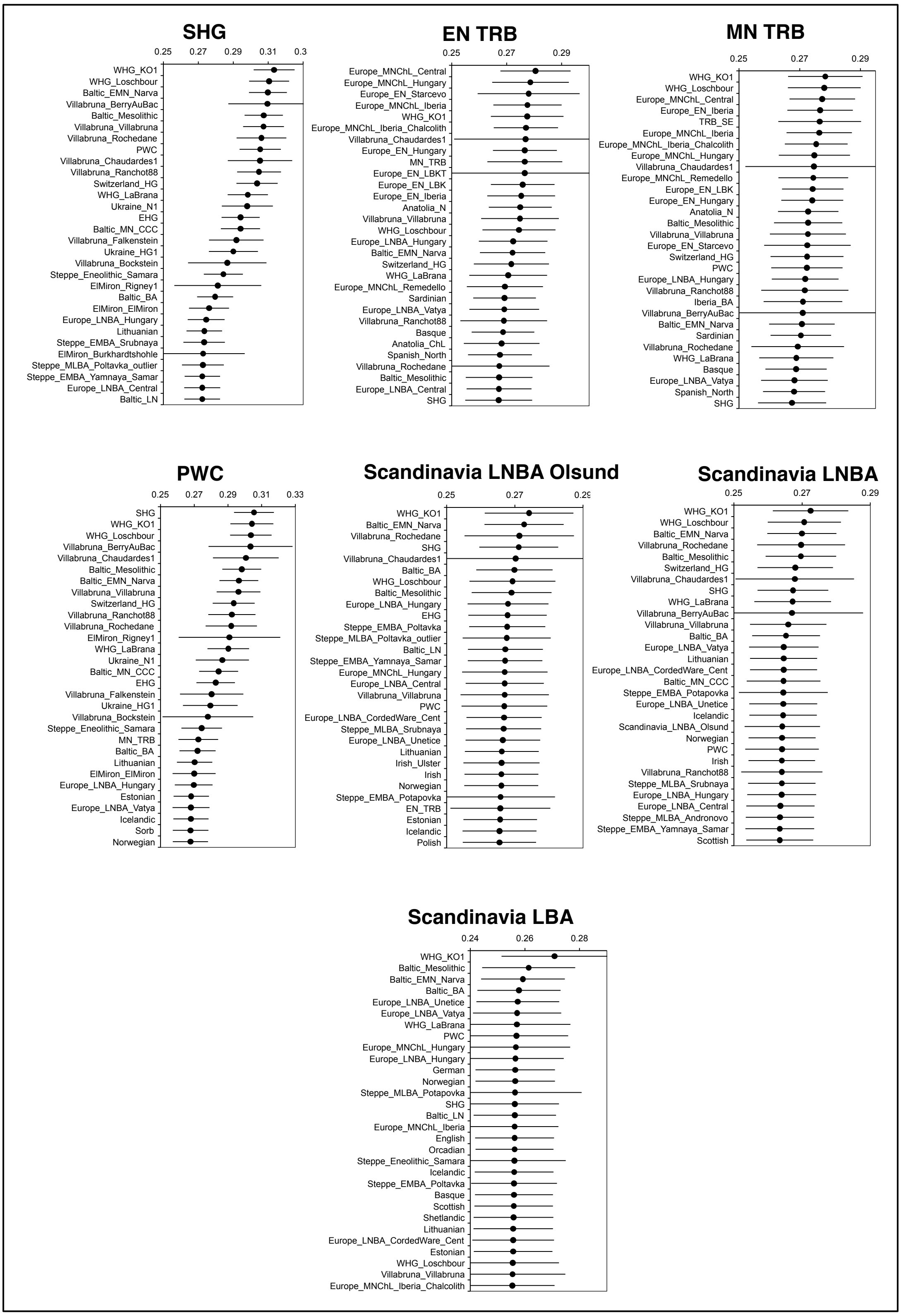

Supplementary Figure 3: Outgroup f_3-statistics for ancient Scandinavian populations. Values shown for the statistic f_3(Ancient Scandinavian Population, X; Mbuti), where X is a modern or ancient population. The thirty highest hits are shown and error bars represent three standard errors.

that with EHG.

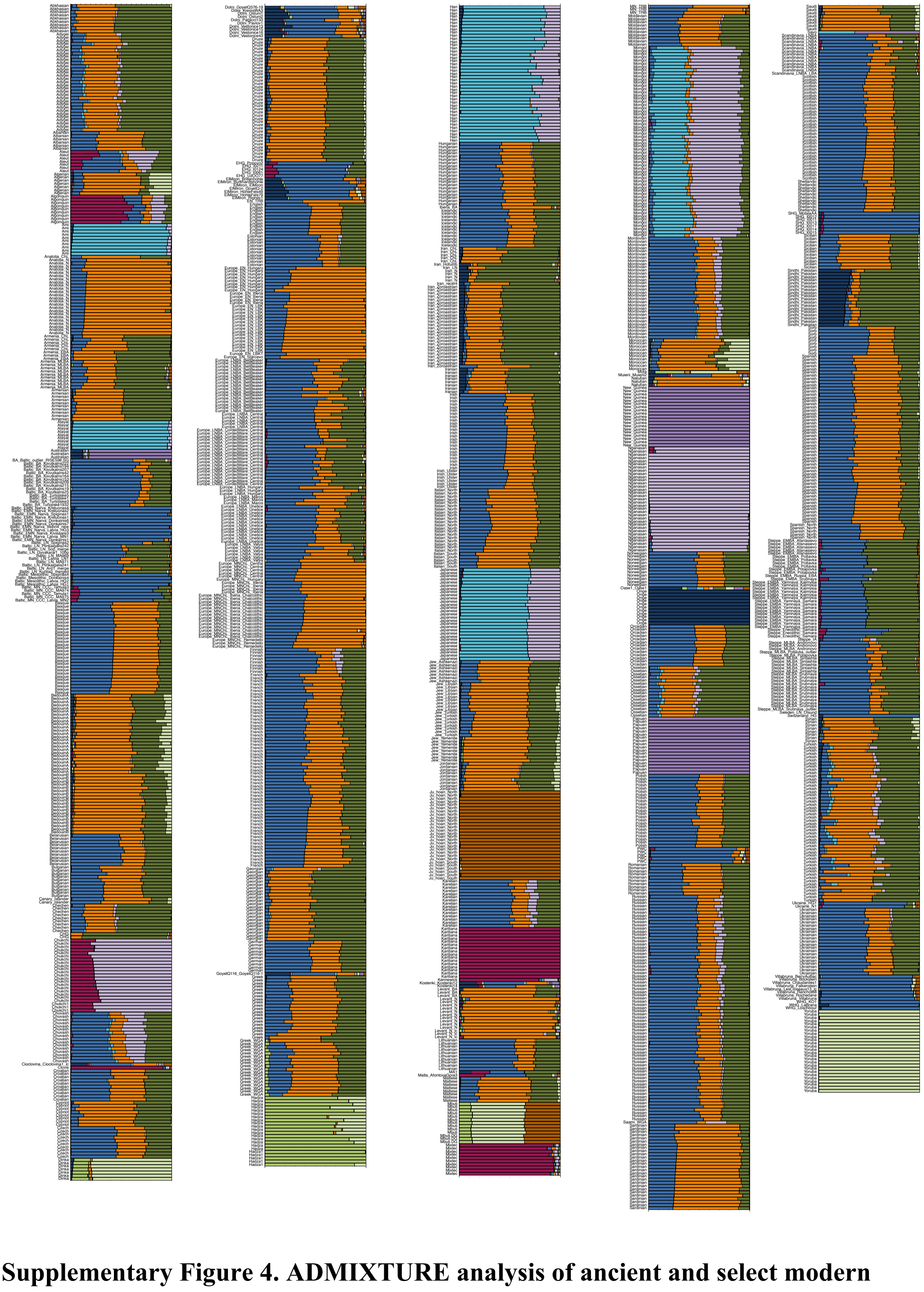

Supplementary Figure 4. ADMIXTURE analysis of ancient and select modern individuals at k=11.

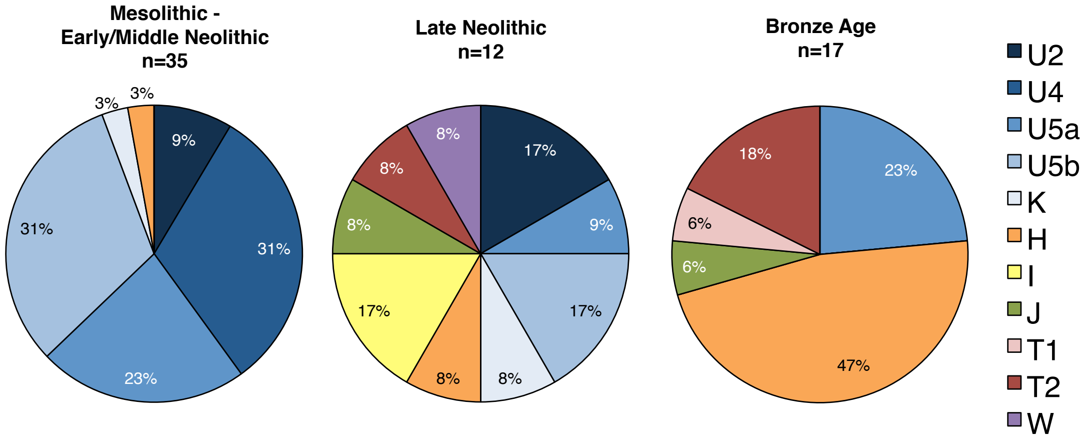

Supplementary Figure 5: Shift of mtDNA haplogroup frequencies at the onset of the Late Neolithic in the Eastern Baltic region. Frequency pie charts of 53 Eastern Baltic mtDNA haplogroups generated in this study and eleven haplogroups from Bramanti et al. (2009), Jones et al. (2017) and Saag et al. (2017).

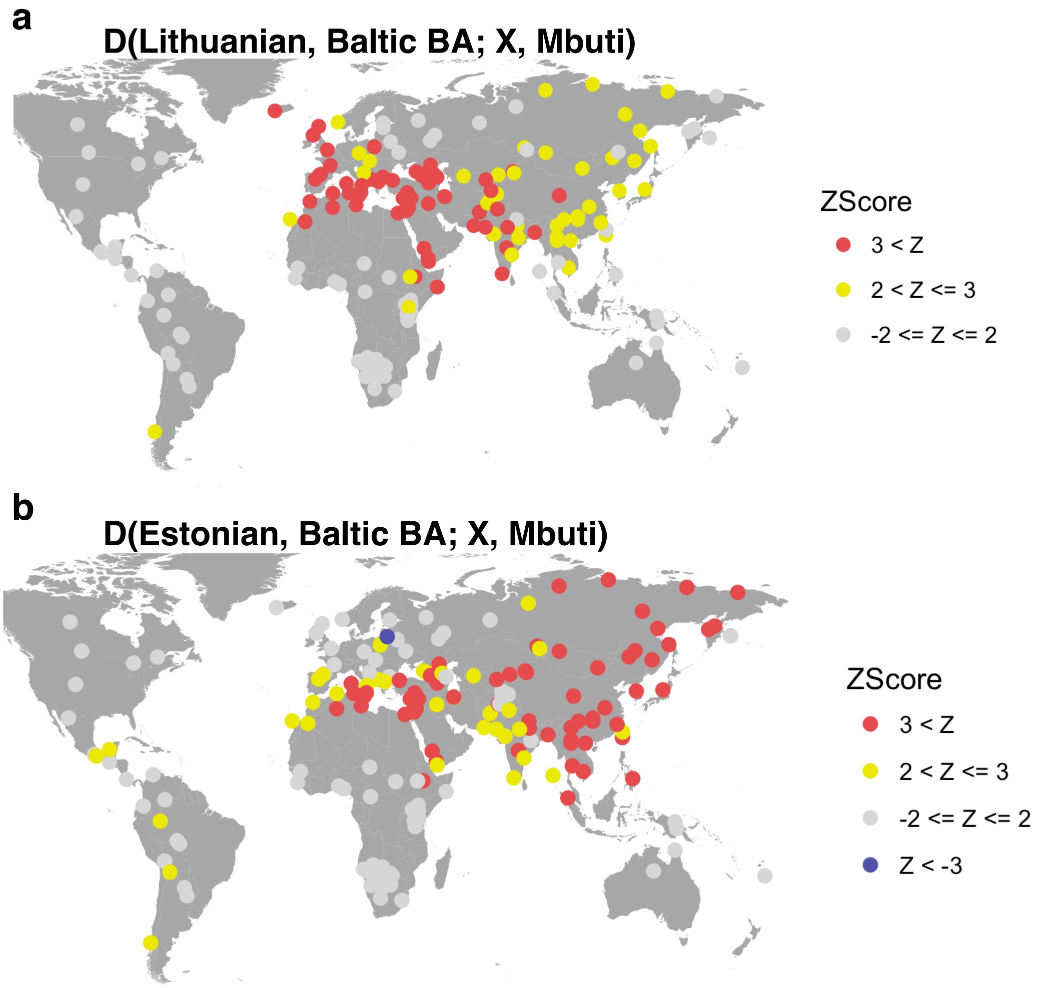

Supplementary Figure 6. D-statistics showing affinities of modern Eastern Baltic populations. Heatmap codes significance of excess allele sharing of modern **(a)** Lithuanians and **(b)** Estonians with modern worldwide populations when compared with Baltic BA. Maps made with R 3.2.3[168] (http://www.R-project.org) package *ggplot2*[169] (https://cran.r-project.org/web/packages/ggmap/citation.html) and package *ggmap*[170] (https://cran.r-project.org/web/packages/ggplot2/citation.html).

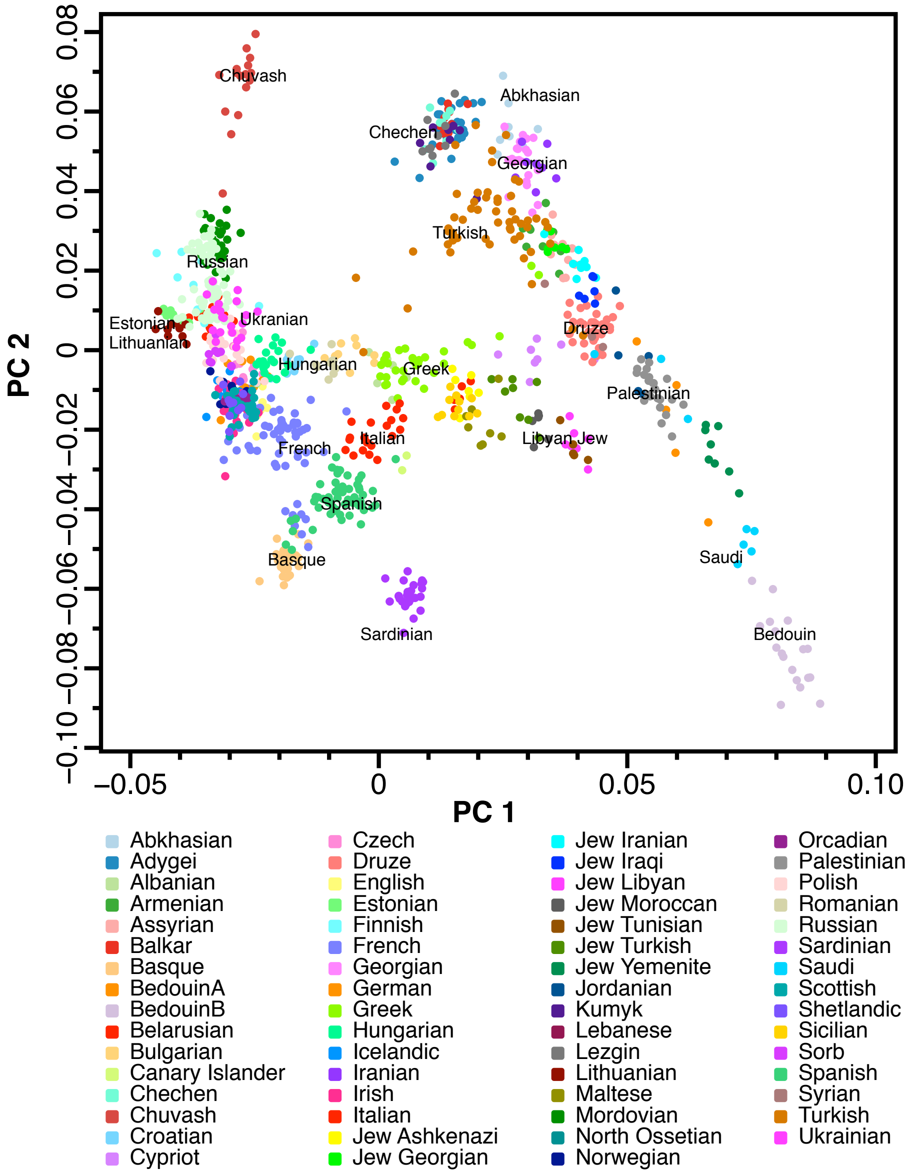

Supplementary Figure 7: PCA analysis on modern West Eurasian populations. Showing population labels for 1007 modern individuals used to construct the PCA in Fig. 2a.

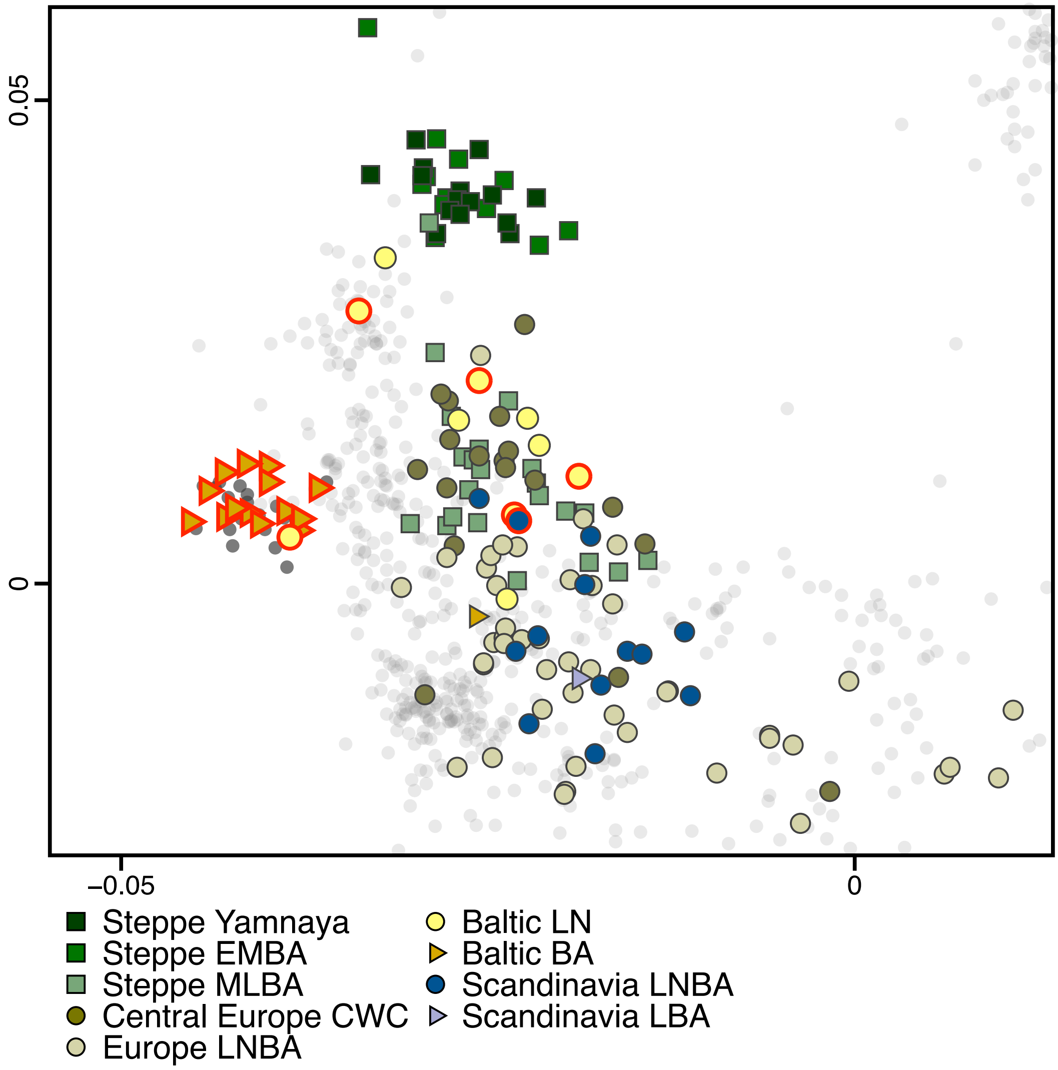

Supplementary Figure 8: PCA projection of Late Neolithic and Bronze Age Europeans. Zoom-in of corresponding plot in Fig. 2a.

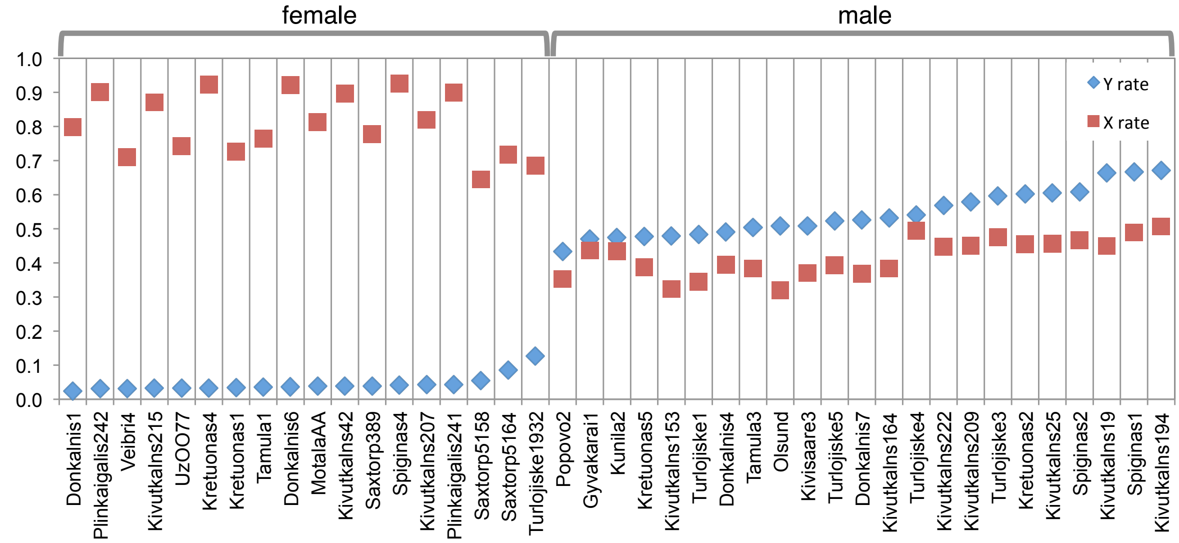

Supplementary Figure 9: Sex determination using the X rate/Y rate method. The Y rate shows greater usefulness in distinguishing male from female samples.

Supplementary Table 1: Negative results for admixture f_3(Source A, Source B; Target). All combinations of ancient populations were tested as sources for a Target ancient Scandinavian or Eastern Baltic population under study. The ten most negative hits are shown for tests with more negative results.

Target	Source A	Source B	f_3-value	Std. error	Z
EHG	Baltic_Mesolithic	AfontovaGora3	-0.013151	0.002547	-5.163
	AfontovaGora3	Switzerland_HG	-0.012441	0.003083	-4.035
	Baltic_EMN_Narva	AfontovaGora3	-0.012177	0.002256	-5.397
	AfontovaGora3	PWC	-0.01141	0.002815	-4.053
	AfontovaGora3	SHG	-0.011204	0.002363	-4.742
	AfontovaGora3	Ukraine_HG1	-0.007924	0.00594	-1.334
	Europe_LNBA_Maros	AfontovaGora3	-0.00611	0.003914	-1.561
	AfontovaGora3	EN_TRB	-0.005882	0.00347	-1.695
	Europe_MNChL_Hungary	AfontovaGora3	-0.005723	0.00304	-1.883
	Europe_MNChL_Central	AfontovaGora3	-0.005499	0.002712	-2.028
Baltic Mesolithic	Steppe_Eneolithic_Samara	Switzerland_HG	-0.014182	0.002178	-6.513
	AfontovaGora3	Switzerland_HG	-0.014111	0.003028	-4.66
	AG2	Switzerland_HG	-0.013876	0.004161	-3.335
	EHG	Switzerland_HG	-0.013241	0.00205	-6.459
	Baltic_MN_CCC	Switzerland_HG	-0.010459	0.002238	-4.674
	Steppe_EMBA_Srubnaya	Switzerland_HG	-0.009902	0.002852	-3.472
	Switzerland_HG	Ukraine_N1	-0.008816	0.003598	-2.45
	Steppe_MLBA_Potapovka	Switzerland_HG	-0.008308	0.0027	-3.078
	Steppe_EMBA_Potapovka	Switzerland_HG	-0.008118	0.002913	-2.787
	Clovis	Switzerland_HG	-0.007863	0.002489	-3.158
Baltic MN CCC	AfontovaGora3	Switzerland_HG	-0.022033	0.004049	-5.442
	Europe_EN_Starcevo	AfontovaGora3	-0.018078	0.007006	-2.58
	Baltic_Mesolithic	AfontovaGora3	-0.015762	0.003237	-4.87
	Baltic_EMN_Narva	AfontovaGora3	-0.015625	0.002952	-5.293
	AfontovaGora3	PWC	-0.011136	0.003811	-2.922
	Europe_MNChL_Hungary	AfontovaGora3	-0.010885	0.003943	-2.761
	AfontovaGora3	SHG	-0.010669	0.003074	-3.471
	AfontovaGora3	MN_TRB	-0.008514	0.004338	-1.963
	AfontovaGora3	EN_TRB	-0.007921	0.005089	-1.556
	Europe_MNChL_Iberia	AfontovaGora3	-0.00771	0.003254	-2.369
Baltic LNBA	Europe_EN_LBK	AfontovaGora3	-0.016595	0.001217	-13.639
	Europe_EN_Iberia	AfontovaGora3	-0.016528	0.001566	-10.551
	Europe_MNChL_Hungary	AfontovaGora3	-0.015738	0.002046	-7.691
	AfontovaGora3	MN_TRB	-0.015693	0.002058	-7.624
	Europe_MNChL_Central	AfontovaGora3	-0.015653	0.001737	-9.013
	Europe_EN_Starcevo	AfontovaGora3	-0.015491	0.003445	-4.497
	Europe_EN_Hungary	AfontovaGora3	-0.015352	0.001257	-12.211
	Anatolia_N	AfontovaGora3	-0.014881	0.001193	-12.474
	Europe_MNChL_Iberia	AfontovaGora3	-0.014745	0.001534	-9.611
	Satsurblia_Kotias	Ukraine_N1	-0.0142	0.002403	-5.91
Baltic BA	Baltic_Mesolithic	Levant_BA	-0.006667	0.000972	-6.861
	Baltic_EMN_Narva	Iran_ChL	-0.005876	0.000738	-7.958
	Europe_MNChL_Hungary	AfontovaGora3	-0.005778	0.002013	-2.871
	Europe_EN_LBK	AfontovaGora3	-0.005742	0.001223	-4.696
	Levant_BA	Switzerland_HG	-0.005721	0.001291	-4.43
	Baltic_EMN_Narva	Iran_LN	-0.005561	0.001088	-5.11
	Europe_EN_Iberia	AfontovaGora3	-0.005511	0.001583	-3.48
	Iran_LN	Switzerland_HG	-0.005344	0.001838	-2.908
	Anatolia_ChL	Switzerland_HG	-0.005037	0.001816	-2.773
	Baltic_EMN_Narva	Iran_N	-0.004951	0.00101	-4.902
MN TRB	Scandinavia_LNBA	EN_TRB	-0.005415	0.006671	-0.812
	Natufian	Switzerland_HG	-0.021067	0.006912	-3.048
	Levant_N	Switzerland_HG	-0.019363	0.005397	-3.588
	Natufian	PWC	-0.018439	0.00683	-2.7
	Baltic_Mesolithic	Natufian	-0.016706	0.005976	-2.796
	Iran_LN	Switzerland_HG	-0.014486	0.007316	-1.98
	Anatolia_N	Switzerland_HG	-0.013243	0.004	-3.311
	Europe_EN_LBK	Switzerland_HG	-0.012758	0.004164	-3.064
	Anatolia_ChL	Switzerland_HG	-0.010833	0.006861	-1.579
Scandinavia LNBA	Europe_EN_Starcevo	AfontovaGora3	-0.01553	0.003932	-3.949
	Europe_EN_Iberia	AfontovaGora3	-0.01516	0.001833	-8.271
	Europe_EN_LBK	AfontovaGora3	-0.014564	0.001474	-9.88
	Europe_MNChL_Iberia	AfontovaGora3	-0.01405	0.001748	-8.039
	Europe_EN_Hungary	AfontovaGora3	-0.01364	0.00152	-8.975
	Anatolia_ChL	Switzerland_HG	-0.013443	0.002069	-6.496
	Europe_MNChL_Central	AfontovaGora3	-0.01339	0.002111	-6.342
	Anatolia_N	AfontovaGora3	-0.013065	0.00144	-9.075
	AfontovaGora3	MN_TRB	-0.013047	0.002357	-5.535
	Levant_BA	Switzerland_HG	-0.012884	0.001444	-8.922

Supplementary Table 2: Significantly positive results for *D*(*Test=Ancient Eastern Baltic population*, *A*; *B*, Mbuti). *A* is a genetically proximate population to *Test* and *B* is iterated over every other ancient population. This test shows if an *Ancient Eastern Baltic Test population* shares significantly more alleles with *B* than *A* does with *B* when Z>3.

Test	*A*	*B*	*D*	Z
**EHG**	WHG	Malta_AfontovaGora3	0.1152	22.396
		MA1	0.0651	13.62
		Steppe_Eneolithic_Samara	0.0595	15.294
		Steppe_EMBA_Srubnaya	0.0544	10.42
		Clovis	0.0512	11.573
		Kennewick	0.0507	10.753
		AG2	0.046	6.548
		Baltic_MN_CCC	0.039	9.893
		Steppe_EMBA_Yamnaya_Kalmykia	0.0291	9.354
		Steppe_EMBA_Poltavka	0.0274	8.072
		Iran_HotuIIIb	0.0271	4.672
		Steppe_EMBA_Afanasievo	0.026	7.529
		Steppe_EMBA_Yamnaya_Samara	0.0259	8.428
		Steppe_EMBA_Potapovka	0.0259	4.76
		Ukraine_N1	0.0243	3.924
		Steppe_EMBA_Russia_EBA	0.0197	3.503
		Steppe_IA	0.017	3.855
		Iran_N	0.0123	3.412
**Baltic Mesolithic**	WHG	AfontovaGora3	0.024	4.873
		Ukraine_N1	0.0221	4.071
		EHG	0.0203	6.205
		Baltic_MN_CCC	0.0173	4.679
		MA1	0.0165	3.841
		Steppe_Eneolithic_Samara	0.0162	4.659
		Steppe_EMBA_Srubnaya	0.0143	3.366
		Steppe_EMBA_Yamnaya_Kalmykia	0.0111	4.156
		Steppe_EMBA_Poltavka	0.0098	3.351
		Steppe_EMBA_Afanasievo	0.0095	3.185
**Baltic EMN Narva**	WHG	AfontovaGora3	0.0287	7.162
		EHG	0.0204	7.37
		Ukraine_N1	0.018	3.784
		Iran_HotuIIIb	0.017	3.741
		Steppe_Eneolithic_Samara	0.015	5.058
		Baltic_MN_CCC	0.0148	5.063
		Steppe_EMBA_Srubnaya	0.013	3.509
		Steppe_EMBA_Poltavka	0.0127	5.154
		MA1	0.0127	3.634
		Steppe_EMBA_Afanasievo	0.011	4.561
		Sweden_LN_Olsund	0.011	3.435
		Steppe_EMBA_Yamnaya_Kalmykia	0.0108	4.569
		Steppe_EMBA_Yamnaya_Samara	0.0102	4.476
		Baltic_LN	0.0086	4.14
		Baltic_BA	0.0077	3.672
		Europe_LNBA_CordedWare_Central	0.0075	3.479
		Armenia_ChL	0.0072	3.185
		Steppe_MLBA_Srubnaya	0.0065	3.074
**Baltic MN CCC**	EHG	Switzerland_HG	0.0172	3.643
		WHG	0.0134	3.552
		MN_TRB	0.0133	3.092
**Baltic LN**	CordedWare_Central	Baltic_MN_CCC	0.007	3.066
**Baltic LN**	Yamnaya_Samara	Europe_EN_Starcevo	0.0118	3.478
		Europe_MNChL_Hungary	0.0106	4.356
		Switzerland_HG	0.0098	3.903
		Europe_MNChL_Central	0.0087	4.224
		Europe_EN_Iberia	0.0084	4.306
		Europe_MNChL_Remedello	0.008	3.376
		Europe_EN_Hungary	0.0077	4.59
		Europe_MNChL_Iberia_Chalcolithic	0.0077	4.513
		Levant_N	0.0075	3.788
		Baltic_Mesolithic	0.0074	3.297
		Europe_MNChL_Iberia	0.007	3.745
		Europe_EN_LBK	0.0057	3.326
		Anatolia_N	0.0054	3.329
**Baltic BA**	BalticLN	WHG	0.0269	13.218
		Baltic_EMN_Narva	0.0255	13.967
		Switzerland_HG	0.0231	9.143
		Europe_LNBA_Hungary	0.0193	9.778
		Europe_EN_Hungary	0.0192	11.83
		Baltic_Mesolithic	0.0192	8.993
		Europe_MNChL_Iberia	0.0182	10.024
		Europe_MNChL_Iberia_Chalcolithic	0.0168	10.331
		Europe_EN_LBK	0.0162	9.894
		Europe_EN_LBKT	0.0162	3.017
		Anatolia_N	0.016	9.957
		Europe_EN_Starcevo	0.012	3.26

Supplementary Table 3: *qpWave/qpAdm* analysis of possible waves of admixture and admixture coefficients. Tests that are rejected (p<0.05) are marked in red.

Left Population A	Left Population B	Left Population C	Tested rank	Rank p-value	Admixture coefficent for Left Population A		Std. error	
					B	C	B	C
EHG	WHG		0	5.10E-190				
SHG	WHG		0	6.79E-60				
SHG	EHG	WHG	1	1.93E-01	0.43	0.57	0.02	0.02
Baltic_Mesolithic_Donkalnis4	WHG		0	7.95E-01				
Baltic_Mesolithic_Donkalnis4	EHG	WHG	1	7.38E-01	infeasible			
Baltic_Mesolithic_Spiginas4	WHG		0	5.44E-02				
Baltic_Mesolithic_Spiginas4	EHG	WHG	1	2.71E-01	0.09	0.91	0.04	0.04
Latvia_HG1	WHG		0	1.49E-16				
Latvia_HG1	EHG	WHG	1	1.47E-01	0.32	0.68	0.03	0.03
Latvia_HG2	WHG		0	1.45E-13				
Latvia_HG2	EHG	WHG	1	3.98E-01	0.28	0.72	0.03	0.03
Baltic_EMN_Narva_Donkalnis1	WHG		0	3.77E-01				
Baltic_EMN_Narva_Donkalnis1	EHG	WHG	1	2.98E-01	0.04	0.96	0.08	0.08
Baltic_EMN_Narva_Donkalnis6	WHG		0	6.04E-01				
Baltic_EMN_Narva_Donkalnis6	EHG	WHG	1	6.17E-01	0.04	0.96	0.03	0.03
Baltic_EMN_Narva_Donkalnis7	WHG		0	2.08E-11				
Baltic_EMN_Narva_Donkalnis7	EHG	WHG	1	1.21E-01	0.28	0.72	0.04	0.04
Baltic_EMN_Narva_Kivisaare3	WHG		0	1.39E-05				
Baltic_EMN_Narva_Kivisaare3	EHG	WHG	1	9.86E-01	0.28	0.72	0.05	0.05
Baltic_EMN_Narva_Kretuonas1	WHG		0	6.13E-05				
Baltic_EMN_Narva_Kretuonas1	EHG	WHG	1	2.28E-01	0.19	0.81	0.04	0.04
Baltic_EMN_Narva_Kretuonas2	WHG		0	1.12E-05				
Baltic_EMN_Narva_Kretuonas2	EHG	WHG	1	1.27E-01	0.17	0.83	0.03	0.03
Baltic_EMN_Narva_Kretuonas4	WHG		0	7.45E-03				
Baltic_EMN_Narva_Kretuonas4	EHG	WHG	1	8.90E-01	0.14	0.86	0.03	0.03
Baltic_EMN_Narva_Kretuonas5	WHG		0	9.80E-05				
Baltic_EMN_Narva_Kretuonas5	EHG	WHG	1	5.23E-01	0.23	0.77	0.05	0.05
Baltic_EMN_Narva_Spiginas1	WHG		0	8.47E-02				
Baltic_EMN_Narva_Spiginas1	EHG	WHG	1	3.03E-01	0.08	0.92	0.03	0.03
Baltic_EMN_Narva_Veibri4	WHG		0	5.49E-24				
Baltic_EMN_Narva_Veibri4	EHG	WHG	1	5.54E-01	0.43	0.57	0.04	0.04
Latvia_HG3	WHG		0	1.61E-09				
Latvia_HG3	EHG	WHG	1	4.97E-01	0.28	0.72	0.04	0.04
Latvia_MN1	WHG		0	1.02E-07				
Latvia_MN1	EHG	WHG	1	8.04E-01	0.35	0.65	0.05	0.05
Baltic_MN_CCC_Tamula1	EHG		0	1.24E-04				
Baltic_MN_CCC_Tamula1	EHG	WHG	1	1.88E-01	0.73	0.27	0.06	0.06
Baltic_MN_CCC_Tamula3	EHG		0	9.63E-03				
Baltic_MN_CCC_Tamula3	EHG	WHG	1	6.27E-01	0.76	0.24	0.06	0.06
Latvia_MN2	EHG		0	3.06E-01				
Latvia_MN2	EHG	WHG	1	4.08E-01	0.94	0.06	0.04	0.04
CCC_Kud2	EHG		0	6.63E-03				
CCC_Kud2	EHG	WHG	1	2.75E-02	0.77	0.23	0.10	0.10
CCC_Kud2	EHG	SHG	1	6.08E-01	0.84	0.16	0.12	0.12
CCC_Kud3	EHG		0	4.81E-01				
CCC_Kud3	EHG	WHG	1	5.10E-01	0.91	0.09	0.08	0.08
LBK	Loschbour		0	1.34E-212				
EN TRB	LBK	Loschbour	1	2.57E-01	0.81	0.19	0.03	0.03
LBK	KO1		0	5.98E-149				
EN TRB	LBK	KO1	1	2.07E-01	0.76	0.24	0.04	0.04
LBK	LaBrana		0	1.41E-111				
EN TRB	LBK	LaBrana	1	1.84E-01	0.72	0.28	0.05	0.05
LBK	Baltic Mesolithic		0	5.02E-223				
EN TRB	LBK	Baltic Mesolithic	1	6.05E-02	0.75	0.25	0.04	0.04
LBK	Baltic EMN Narva		0	0				
EN TRB	LBK	Baltic EMN Narva	1	1.25E-01	0.75	0.25	0.04	0.04
LBK	SHG		0	3.22E-224				
EN TRB	LBK	SHG	1	3.76E-03	0.76	0.24	0.05	0.05
EN TRB	Europe MNChL Central		0	4.56E-01				
PWC	SHG		0	1.48E-06				
PWC	SHG	EN TRB	1	3.08E-01	0.74	0.26	0.06	0.06

Supplementary Table 4: Significantly positive results for *D*(*Test*=*Ancient Scandinavian population*, A;*X*, Mbuti). *A* is a genetically proximate population to *Test* and *B* is iterated over every other ancient population. This test shows if an *Ancient Scandinavian population* shares significantly more alleles with *B* than *A* does with *B* when *Z*>3.

Test	*A*	*B*	*D*	Z
**SHG**	WHG	AfontovaGora3	0.0273	5.795
		PWC	0.0248	6.657
		EHG	0.0231	7.339
		MA1	0.0188	4.625
		Steppe_Eneolithic_Samara	0.0175	5.279
		Clovis	0.0144	3.766
		Steppe_EMBA_Srubnaya	0.0139	3.228
		Steppe_EMBA_Yamnaya_Samara	0.0137	5.338
		Kennewick	0.0129	3.093
		Steppe_EMBA_Poltavka	0.012	4.043
		Iran_N	0.0119	3.491
		Levant_BA	0.0113	4.164
		Baltic_MN_CCC	0.0107	3.067
		Armenia_ChL	0.0096	3.488
		Steppe_EMBA_Yamnaya_Kalmykia	0.0089	3.294
		Europe_LNBA_Central	0.0084	3.158
		Europe_LNBA_CordedWare_Central	0.0079	3.024
**PWC**	SHG	No results Z>3		
**EN_TRB**	Europe_EN_LBK	Switzerland_HG	0.0364	7.268
		WHG	0.0295	7.561
		Baltic_EMN_Narva	0.0292	8.597
		Baltic_Mesolithic	0.0251	6.576
		SHG	0.0223	6.316
		PWC	0.0182	4.154
		Baltic_MN_CCC	0.0174	4.434
		EHG	0.0165	4.6
		Baltic_BA	0.0137	4.702
		Steppe_Eneolithic_Samara	0.0129	3.267
**EN_TRB**	Europe_MNChL_Central	No results Z>3		
**MN_TRB**	EN_TRB	No results Z>3		
**MN_TRB**	Europe_MNChL_Central	PWC	0.0221	4.631
**Scandinavia LNBA**	Baltic_LN	Europe_EN_Iberia	0.0125	5.36
		Europe_MNChL_Remedello	0.0113	3.963
		Europe_EN_LBK	0.0106	5.369
		Europe_MNChL_Iberia	0.0106	4.84
		MN_TRB	0.0101	3.747
		EN_TRB	0.0098	3.351
		Anatolia_N	0.0095	5.018
		Europe_EN_Hungary	0.0095	4.865
		Europe_MNChL_Iberia_Chalcolithic	0.0083	4.111
		Levant_N	0.0081	3.344
		Europe_MNChL_Central	0.0081	3.19
**Scandinavia LNBA**	Europe LNBA Corded Ware Central	No results Z>3		
**Scandinavia LNBA Olsund**	Baltic_LN	Levant_BA	0.0183	5.073
		Anatolia_ChL	0.0164	3.398
		Levant_N	0.0156	3.913
		Anatolia_N	0.0131	4.024
		Europe_EN_LBK	0.0128	3.81
		Armenia_EBA	0.0127	3.63
		Europe_MNChL_Iberia	0.0118	3.106
		Europe_EN_Hungary	0.0103	3.005
		Europe_MNChL_Iberia_Chalcolithic	0.0101	3.011
**Scandinavia LNBA Olsund**	Scandinavia LNBA	Levant_BA	0.0106	3.082

Supplementary Table 5: Significantly positive results for *D*(*Test=Baltic Neolithic HG*, A=WHG; *B*, Mbuti), testing individuals. *B* is iterated over every other ancient population. This test shows if a *Baltic Neolithic HG* shares significantly more alleles with *B* than WHG does with *B* when Z>3.

Test	*A*	*B*	*D*	Z
Baltic_EMN_Narva_Donkalnis1	WHG	No results Z>3		
Baltic_EMN_Narva_Donkalnis6	WHG	EHG	0.0129	3.035
		Europe_LNBA_Laz_CordedWare_Central	0.0109	3.293
		Baltic_LN	0.0103	3.119
Baltic_EMN_Narva_Donkalnis7	WHG	Malta_AfontovaGora3	0.0472	6.335
		EHG	0.0283	6.292
		MA1	0.0236	3.832
		Steppe_Eneolithic_Samara	0.0197	3.862
		Steppe_EMBA_Poltavka	0.0175	4.285
		Iran_N	0.0148	3.141
		Steppe_EMBA_Yamnaya_Samara	0.0137	3.759
		Steppe_EMBA_Yamnaya_Kalmykia	0.0132	3.362
		Armenia_ChL	0.0113	3.094
		Europe_LNBA_CordedWare_Central	0.0109	3.115
		Baltic_LN	0.0105	3.019
Baltic_EMN_Narva_Kivisaare3	WHG	EHG	0.0193	3.388
Baltic_EMN_Narva_Kretuonas1	WHG	Steppe_EMBA_Potapovka	0.0255	3.018
		EHG	0.0185	3.866
		Steppe_Eneolithic_Samara	0.0184	3.317
		Steppe_EMBA_Yamnaya_Samara	0.015	3.853
		Steppe_EMBA_Poltavka	0.0143	3.266
Baltic_EMN_Narva_Kretuonas2	WHG	EHG	0.0161	3.707
		Steppe_EMBA_Poltavka	0.0118	3.039
Baltic_EMN_Narva_Kretuonas4	WHG	Malta_AfontovaGora3	0.0293	4.542
		EHG	0.0217	5.175
		Steppe_Eneolithic_Samara	0.015	3.195
Baltic_EMN_Narva_Kretuonas5	WHG	Malta_AfontovaGora3	0.0351	3.5
Baltic_EMN_Narva_Spiginas1	WHG	Malta_AfontovaGora3	0.0227	3.301
		Steppe_MLBA_Poltavka_outlier	0.0164	3.245
		Iran_LN	0.0161	3.029
		Steppe_EMBA_Poltavka	0.0142	3.757
		Baltic_BA	0.0119	3.567
		Europe_LNBA_CordedWare_Central	0.0102	3.052
Baltic_EMN_Narva_Veibri4	WHG	Malta_AfontovaGora3	0.0516	6.854
		EHG	0.042	8.892
		Steppe_EMBA_Srubnaya	0.0315	4.601
		Steppe_Eneolithic_Samara	0.0311	5.982
		MA1	0.0285	4.672
		Kennewick	0.0239	3.811
		Clovis	0.0209	3.834
		Steppe_EMBA_Poltavka	0.0203	4.893
		Steppe_EMBA_Yamnaya_Samara	0.0196	4.99
		Steppe_EMBA_Afanasievo	0.0179	4.078
		Steppe_EMBA_Yamnaya_Kalmykia	0.0172	4.379
		Iran_N	0.0158	3.146
Baltic_MN_CCC_Tamula1	WHG	Malta_AfontovaGora3	0.0731	6.504
		EHG	0.0452	7.451
		MA1	0.0394	4.871
		Steppe_EMBA_Srubnaya	0.0382	3.726
		Steppe_Eneolithic_Samara	0.0363	5.169
		Clovis	0.0294	4.233
		Iran_N	0.0218	3.274
		Steppe_EMBA_Poltavka	0.0215	4.02
		Steppe_EMBA_Yamnaya_Samara	0.0213	4.3
		Satsurblia_Kotias	0.0213	3.27
		Steppe_EMBA_Afanasievo	0.0202	3.397
		Steppe_EMBA_Yamnaya_Kalmykia	0.0188	3.652
Baltic_MN_CCC_Tamula3	WHG	Malta_AfontovaGora3	0.0938	9.54
		AG2	0.0636	3.518
		Steppe_EMBA_Srubnaya	0.0554	5.664
		EHG	0.0539	8.929
		Steppe_Eneolithic_Samara	0.0533	7.823
		MA1	0.0506	6.508
		Steppe_EMBA_Russia_EBA	0.0473	3.449
		Kennewick	0.0464	5.257
		Clovis	0.0458	6.615
		Steppe_EMBA_Poltavka	0.0336	6.394
		Steppe_EMBA_Afanasievo	0.0316	5.598
		Steppe_EMBA_Yamnaya_Kalmykia	0.0287	5.352
		Steppe_EMBA_Yamnaya_Samara	0.0279	5.786
		Baltic_LN	0.0179	3.872
		Europe_LNBA_CordedWare_Central	0.0146	3.041

Supplementary Table 6: Allele information of SNPs thought to be affected by selection. Bases with q>30 are counted, we report the plus strand. rs4988235 is responsible for lactase persistence in Europe, the SNPs at *SLC24A5* and *SLC45A2* for light skin pigmentation. The SNP at *EDAR* affects tooth morphology and hair thickness. The SNP at *HERC2* is the primary determinant of light eye color in modern Europeans. We highlight likely heterozygous sites in pink and sites that are likely homozygous for the derived allele in blue. DAF= Derived allele frequency.

Population	Sample	SNP	LCT rs4988235	SLC45A2 rs16891982	SLC24A5 rs1426654	EDAR rs3827760	HERC2 rs12913832
		**Ancestral**	**G**	**C**	**G**	**A**	**A**
		**Derived**	**A**	**G**	**A**	**G**	**G**
**EHG**	UzOO77	Coverage	3	1	0	8	2
		DAF	0%	0%	n/a	0%	0%
**Baltic Mesolithic**	Spiginas4	Coverage	4	3	2	0	2
		DAF	0%	0%	0%	n/a	100%
**Baltic EMN Narva**	Spiginas1	Coverage	30	31	6	20	13
		DAF	0%	0%	50%	0%	100%
	Donkalnis6	Coverage	34	17	1	21	12
		DAF	0%	0%	100%	0%	100%
	Donkalnis7	Coverage	3	4	0	1	2
		DAF	0%	0%	n/a	0%	100%
	Kretuonas1	Coverage	1	1	1	1	0
		DAF	0%	0%	100%	0%	n/a
	Kretuonas2	Coverage	4	5	1	6	2
		DAF	0%	100%	100%	0%	100%
	Kretuonas4	Coverage	48	25	4	38	18
		DAF	0%	100%	75%	0%	100%
	Veibri4	Coverage	5	5	0	3	3
		DAF	0%	0%	n/a	0%	0%
	Kivisaare3	Coverage	1	0	0	0	2
		DAF	0%	n/a	n/a	n/a	100%
**Baltic MN CCC**	Tamula1	Coverage	1	1	2	0	1
		DAF	0%	0%	100%	n/a	100%
**Baltic LN**	Gyvakarai1	Coverage	2	4	3	2	5
		DAF	0%	50%	100%	0%	100%
	Kunila2	Coverage	1	0	1	0	1
		DAF	0%	n/a	100%	n/a	0%
	Spiginas1	Coverage	30	31	6	20	13
		DAF	0%	0%	50%	0%	100%
	Plinkaigalis241	Coverage	1	0	0	2	0
		DAF	100%	n/a	n/a	0%	n/a
	Plinkaigalis242	Coverage	7	5	3	9	2
		DAF	0%	0%	100%	0%	0%
**EN TRB**	Kvarlov5164	Coverage	1	2	0	0	0
		DAF	0%	50%	n/a	n/a	n/a
**Sweden LN Olsund**	Olsund	Coverage	9	6	4	9	2
		DAF	0%	0%	100%	0%	0%
**Baltic BA**	Turlojiske3	Coverage	1	0	0	1	2
		DAF	0%	n/a	n/a	0%	100%
	Turlojiske1	Coverage	0	1	0	1	0
		DAF	n/a	100%	n/a	0%	n/a
	Turlojiske5	Coverage	0	1	0	1	1
		DAF	n/a	100%	n/a	0%	100%
	Kivutkalns153	Coverage	2	3	0	2	2
		DAF	0%	100%	n/a	0%	100%
	Kivutkalns207	Coverage	18	28	4	47	15
		DAF	61%	50%	100%	0%	100%
	Kivutkalns19	Coverage	26	19	5	19	14
		DAF	0%	100%	100%	0%	64%
	Kivutkalns194	Coverage	1	0	0	1	0
		DAF	100%	n/a	n/a	0%	n/a
	Kivutkalns215	Coverage	7	11	2	17	3
		DAF	57%	0%	100%	0%	100%
	Kivutkalns209	Coverage	5	11	2	10	3
		DAF	20%	100%	100%	0%	100%
	Kivutkalns25	Coverage	6	6	1	10	1
		DAF	100%	100%	100%	0%	100%
	Kivutkalns222	Coverage	3	4	0	7	3
		DAF	33%	100%	n/a	0%	100%
	Kivutkalns42	Coverage	3	9	0	4	2
		DAF	33%	100%	n/a	0%	0%

Supplementary Table 7: Significantly positive results for *D*(*Test*=*Baltic LN*, A=Yamnaya; *B*, Mbuti), testing individuals. *B* is iterated over every other ancient population. This test shows if a *Baltic LN* shares significantly more alleles with *B* than Yamnaya does with *B* when Z>3. The twelve most positive hits are shown for tests with more results Z>3.

Test	*A*	*B*	*D*	Z
Baltic_LN_Gyvakarai1	Yamnaya Kalmykia/Samara	No results Z>3		
Baltic_LN_Kunila2	Yamnaya Kalmykia	Europe_MNChL_Remedello	0.0222	4.033
		Europe_EN_Iberia	0.02	4.509
		Switzerland_HG	0.0196	3.432
		Natufian	0.0173	3.03
		Europe_MNChL_Iberia_Chalcolithic	0.0171	4.502
		Europe_EN_Hungary	0.017	4.587
		Anatolia_N	0.0146	4.109
		Europe_EN_LBK	0.0136	3.828
		Europe_MNChL_Iberia	0.0135	3.066
	Yamnaya Samara	Europe_EN_Iberia	0.0175	4.059
		Europe_MNChL_Remedello	0.0173	3.317
Baltic_LN_Plinkaigalis241	Yamnaya Kalmykia	EN_TRB	0.0435	5.332
		Europe_MNChL_Central	0.0356	6.172
		Europe_EN_LBK	0.0318	7.65
		Europe_MNChL_Iberia	0.0315	6.256
		Europe_MNChL_Hungary	0.0312	4.781
		Europe_EN_Hungary	0.031	6.886
		Anatolia_N	0.0295	7.14
		Europe_EN_Iberia	0.0294	5.751
		Levant_N	0.0267	4.708
		Europe_MNChL_Iberia_Chalcolithic	0.0262	5.772
		WHG	0.0254	4.673
		Baltic_EMN_Narva	0.0204	4.225
	Yamnaya Samara	EN_TRB	0.0392	5.127
		Europe_EN_Starcevo	0.0338	3.106
		Europe_MNChL_Central	0.0282	5.323
		Europe_MNChL_Iberia	0.0268	5.745
		Europe_MNChL_Hungary	0.0253	4.101
		Europe_EN_LBK	0.0237	5.941
		Europe_EN_Hungary	0.0228	5.387
		Anatolia_N	0.0223	5.681
		Europe_EN_Iberia	0.022	4.584
		Levant_N	0.0217	4.024
		Europe_MNChL_Iberia_Chalcolithic	0.017	4.077
Baltic_LN_Plinkaigalis242	Yamnaya Kalmykia	Baltic_EMN_Narva	0.0172	4.948
		Europe_EN_Hungary	0.0171	5.42
		WHG	0.0155	4.02
		Baltic_BA	0.0151	5.121
		Steppe_MLBA_Poltavka_outlier	0.0151	3.174
		Baltic_Mesolithic	0.0145	3.59
		SHG	0.0137	3.679
		Europe_MNChL_Central	0.0133	3.523
		Anatolia_N	0.0113	3.942
		Europe_MNChL_Iberia_Chalcolithic	0.0106	3.434
		Europe_EN_LBK	0.0098	3.184
	Yamnaya Samara	No results Z>3		
Baltic_LN_Spiginas2	Yamnaya Kalmykia	Switzerland_HG	0.0533	9.841
		WHG	0.0505	11.804
		Baltic_EMN_Narva	0.0471	11.585
		Baltic_Mesolithic	0.0441	10.245
		SHG	0.035	9.013
		Europe_MNChL_Iberia	0.0337	9.215
		Europe_MNChL_Central	0.0323	7.842
		Europe_LNBA_Hungary	0.0316	7.968
		Europe_EN_Hungary	0.0315	9.659
		Europe_MNChL_Iberia_Chalcolithic	0.0312	10.003
		Baltic_BA	0.0303	9.487
		Anatolia_N	0.0252	8.161
	Yamnaya Samara	Switzerland_HG	0.0435	8.442
		WHG	0.0382	8.956
		Baltic_Mesolithic	0.0362	8.593
		Baltic_EMN_Narva	0.0354	8.903
		Europe_MNChL_Iberia	0.0258	7.161
		Europe_MNChL_Iberia_Chalcolithic	0.0239	7.988
		Europe_MNChL_Central	0.0238	6.185
		SHG	0.0229	6.094
		Europe_EN_Hungary	0.0228	7.232
		Baltic_BA	0.0213	6.822
		Europe_EN_Iberia	0.0211	5.872
		Anatolia_N	0.0184	6.132

Supplementary Note 1

Glossary of archaeological abbreviations and terms

We provide a short glossary of abbreviations used in the main text. Please note that some of these have been used widely in the archaeological community (e.g. LBK, TRB, CWC, etc.), whereas other abbreviations have found acceptance in numerous genomic studies of prehistoric individuals (e.g. WHG, EHG, LNBA, etc.).

**AG3**	Afontova Gora 3, Siberia, Upper Paleolithic, 16930–16490 calBP
**ANE**	Ancient North Eurasian; genetic component related to Upper Paleolithic inhabitants of North Eurasia (e.g. MA1, AG3); term coined in Lazaridis et al.[1]
**BA**	Bronze Age, in the Eastern Baltic ca. 1800–500 BCE
**CCC**	Combed Ceramic Culture; Middle Neolithic, pottery-producing hunter-gatherer culture of the Eastern Baltic region, North-Western Russia and Fennoscandia, ca. 4200-1800 BCE
**CHG**	Caucasus Hunter-Gatherers; genetic cluster associated with late Upper Paleolithic and Mesolithic foragers from the Caucasus
**CWC**	Corded Ware Complex, Central and North-Eastern Europe, Late (Final) Neolithic, ca. 2900-2300 BCE
**EHG**	Eastern Hunter-Gatherers, genetic cluster associated with Mesolithic foragers from eastern Europe; term coined in Lazaridis et al.[1]
**EMN**	Early and Middle Neolithic
**EN**	Early Neolithic
**EN TRB**	Early Neolithic Funnel Beaker Culture, earliest farming culture of Southern Scandinavia, ca. 4200–3300 BCE, preceding MN TRB
**Kunda**	Mesolithic hunter-gatherer culture of the Eastern Baltic region, ca. 9000–5000 BCE
**LBK**	Linear pottery culture (from German Linearbandkeramik); Early Neolithic farmers of Central Europe
**LN**	Late Neolithic
**LNBA**	Late Neolithic and Early Bronze Age (ca. 2900-1700 BCE), referring to a transformative period in Europen genetic prehistory that saw the arrival of and continued admixture with a genetic component related to Bronze Age people of the Pontic-Caspian Steppe; term coined in Haak et al.[2]
**MA1**	"Mal'ta boy", Siberia, Upper Paleolithic, 24520–24090 calBP
**MN**	Middle Neolithic
**MN TRB**	Middle Neolithic Funnel Beaker Culture, Southern and Western Sweden, ca. 3300–2900 BCE, succeeding EN TRB and contemporaneous to PWC
**MNChL**	Middle Neolithic and Chalcolithic, farming cultures of Central and Southern Europe
**Narva**	Late Mesolithic to Middle Neolithic, pottery-producing hunter-gatherer culture of the Eastern Baltic region, ca. 5400–1800 BCE

**PWC**	Pitted Ware Culture; coastal hunter-gatherers of Eastern Sweden; ca. 3300–2300 BCE, contemporaneous to MN TRB
**SHG**	Scandinavian Hunter-Gatherers; genetic cluster associated with late Mesolithic foragers from Central Sweden; term coined in Lazaridis et al.[1]
**TRB**	Funnel Beaker Culture (from German Trichterbecher), first farming culture of Scandinavia, ca. 4200–2900 BCE; see EN TRB and MN TRB
**Yamnaya**	Late Copper Age to Early Bronze Age pastoralist culture of the Pontic-Caspian Steppe, ca. 4000–2300 BCE
**WHG**	Western Hunter-Gatherers; genetic cluster associated with Mesolithic foragers from Iberia to Hungary; term coined in Lazaridis et al.[1]

Supplementary Note 2
Archaeological context and sample description

This section provides archeological context for the 24 individuals for whom we report new genome-wide data, as well as the additional 81 human remains that were screened for this study. We also give a general overview of the prehistory of the Eastern Baltic and Scandinavian region as understood through archeology. Uncalibrated radiocarbon dates are in BP, calibrated radiocarbon dates are given at 94.5% confidence interval (calBCE), contextual archeological dating is given in BCE.The radiocarbon dates are not corrected for potential reservoir effects.

A tabular overview of information from all samples and their sequencing results is given in Supplementary Data Table 1.

The Eastern Baltic Region

Despite the similarities in the material culture and the smallness of the region, chronologies of the prehistory in the Baltic States varies, and to understand the context properly these have to be introduced first (Supplementary Table 8).

Supplementary Table 8. Chronology of prehistoric cultures in the Eastern Baltic region. All dates given in BCE.

<table>
<tr><th></th><th colspan="2">Estonia
(after Kriiska[3]; Lang[4])</th><th colspan="2">Latvia
(after Loze[5]; Zagorska[6]; Lang[4])</th><th colspan="2">Lithuania
(after Antanaitis-Jacobs & Girinikas[7]; Antanaitis-Jacobs et al.[8]; Lang[4])</th></tr>
<tr><td rowspan="3">Mesolithic (9000–4300/ 4100)</td><td>Early Mesolithic (9000–7000)</td><td rowspan="2">Kunda Culture (9000–4900)</td><td>Early Mesolithic</td><td>Pulli Culture (9000–6500)</td><td rowspan="2">Early Mesolithic (9000–6000)</td><td rowspan="2">Kunda Culture (9000–5300)</td></tr>
<tr><td rowspan="2">Late Mesolithic (7000–4200)</td><td>Middle Mesolithic</td><td>Kunda Culture (6500–5400)</td></tr>
<tr><td>Narva Culture (4900–4200)</td><td>Late Mesolithic</td><td rowspan="2">Narva Culture (5400–4100)</td><td>Late Mesolithic (6000–4550/4300)</td><td rowspan="4">Narva Culture (5300–1750)

Nemunas Culture (5100 – 3500)</td></tr>
<tr><td rowspan="3">Neolithic (4200–1800)</td><td>Early Neolithic (4200–3600)</td><td>Typical Combed Ware Culture (4200–3600)</td><td>Early Neolithic</td><td>Early Neolithic (4550/4300–3600/3400)</td></tr>
<tr><td rowspan="2">Late Neolithic (3600–1800)</td><td rowspan="2">Late Combed Ware (3600–1800) / Corded Ware (2900–1800)</td><td>Middle Neolithic (4100–2900)</td><td>Combed Ware and East Baltic Ware Cultures</td><td>Middle Neolithic (3600/3400–2400/2300)</td></tr>
<tr><td>Late Neolithic (2900–1800/1500)</td><td>Corded Ware and Abora I Ware Cultures</td><td>Late Neolithic (2400/2300–1800/1500)</td></tr>
<tr><td rowspan="3">Bronze Age (1800–500)</td><td colspan="2">Early Bronze Age (1800 – 1300)</td><td colspan="2">Early Bronze Age (1800/1500–1100)</td><td colspan="2">Early Bronze Age (1800/1500–1300/1100)</td></tr>
<tr><td colspan="2">Middle Bronze Age (1300–900)</td><td colspan="2" rowspan="2">Late Bronze Age (1100–500)</td><td colspan="2" rowspan="2">Late Bronze Age (1300/1100–500)</td></tr>
<tr><td colspan="2">Late Bronze Age (900–500)</td></tr>
</table>

Despite the differences in the dates these chronologies highlight the following archaeologically visible turning points in the prehistory of the Eastern Baltic: (1)

initial colonization of the region; (2) transition of subsistence relying on terrestrial mammals to the sea resources; (3) beginning of pottery production by hunter-gatherers; (4) influx of exotic resources; (5) introduction of farming and crop cultivation; and (6) introduction of first metal objects and metallurgy.

Initial colonization of the Eastern Baltic region occurred after the retreat of the ice sheets in the first half of the 9th millennium BCE and following centuries. The pioneering settlers arrived from south and thus the first settlements are known from the territory of Southern-Lithuania and Latvia (already 11 000 BCE[6,9]) whereas the northern shores of Estonia were inhabited a bit later. During ca. 9000–8500 BCE natural sources of flint, manufacturing techniques and analogous forms of bone and antler artefacts as well as similarities of lithic technologies point to the existence of extensive networks in the European Boreal zone[10]. From ca. 8000 BCE a transition from Paleolithic cultures to the Mesolithic Kunda Culture is seen. The name-giving site Kunda Lammasmägi was discovered already in 1870s during the marl mining[11]. The abundant bone material together with small items from mainly quartz hint on a variety of activities on the site[11,12]. The dominant species was elk[13], while fish and seal bones appeared to be rare, indicating a subsistence dominated by terrestrial mammals[11,13]. In general, Kunda Culture, is characterized by inland camp sites on the shores of larger rivers and lakes, the main pray came from forests – being elk and beaver[9] and the flint technology aiming at using the all the available source material to produce flakes to make small items was prevalent[14]. At the second half of the Kunda period, during the 7th millennium BCE the habitation spread to coast and further to the islands of the Baltic Sea[15]. During the Littorina Sea phase in the Baltic Sea basin hunting of marine mammals became important. From the beginning of the period there are no burial sites known; the earliest human remains – from the end of Kunda period – are known from the Zvejnieki cemetery in Northern Latvia[16,17].

The beginning of pottery production at the region dates back to the 6th millennium BCE being a cultural loan and an invention of hunter-gatherers. This period is named after the sites discovered in Narva area at the North-Eastern Estonia. As the successor of the Kunda Culture, the Narva Culture emerged around ca. 5500 BCE in the eastern inland Baltic region and spread north-, south and coastward in the following millennium, occupying the same region as the Kunda Culture[18]. In Estonia Narva Culture is distinguished only in the Late Mesolithic phase, whereas both in Latvia and Lithuania it continues to the Neolithic (Supplementary Note 1 Table 1). Despite this technological innovation of pottery production there were no marked changes in the life ways or usages of raw materials of hunter-gatherers in the Eastern Baltic region. Continuity with the Mesoltihic tradition is seen in production and use of bone and antler artefacts. Also continued is the subsistence strategy of foraging, and numerous finds show evidence fore reliance on fishing[19,20]. In Estonia the majority of the sites are located on the coast[2] and seal-hunting grew in importance, with presumably specialized seasonal sites for this puropose found on Estonian islands[21]. In the Middle Neolithic first sparse evidence is found in Narva sites for domesticated plants and

animals but foraging ramins the dominant economy[20]. Assemblages of the Narva Culture persist in its southern occupational area until the Late Neolithic[7].

The chronologically and geographically overlapping Combed Ware Culture of pottery producing hunter-gatherers to the north exhibited geographically extensive and intensive spread of foreign materials and artefacts, especially during Early Neolithic. On the territory of present-day Estonia flint, amber and metatuff from foreign sources were widely used. For example, flint is most likely from the upper reaches of the Volga River, but also from South Lithuania or Belarus. The origin of amber is difficult to detect, because the material and form are similar over an extensive area, most likely the amber came from Western Latvia and Western Lithuania. Metatuff used during the Stone Age on the territory of present day Estonia most likely came from the Onega region in Karelia in present day Russia; the most numerous artefact made of metatuff is the Russian-Karelian type of wood chopping tool[10].

The process of Neolithization is different in the Eastern Baltic region from what is known in Central and Southern Europe, where the 'Neolithic package' is mostly defined by simultaneous emergence of pottery production, crop cultivation and herding of domesticated animals, sedentary life way, and monumental architecture. In the Eastern Baltic, it has been a long tradition that the pottery production solely defines the beginning of Neolithic, whereas the life ways of the people do not change that markedly. Recently, however, Kerkko Nordqvist and Aivar Kriiska have listed the characteristics of Neolithization in the region as the following[22]:

- Pottery – great local variability of ceramics, which exhibit further a mixture of local traditions, domestic innovations, and external influences. Many new lines of development, like cultivation and rock art, appear roughly simultaneously with the adoption of pottery, even if these phenomena become more pronounced or visible only much later, especially around and after 4000 calBCE.

- From the 5th millennium calBCE onwards the material culture and the variety of raw materials become increasingly versatile. The broadening selection of mineral raw materials is especially striking. The working of stone materials and, consequently, the properties of artefacts start to change as well.

- The large-scale appearance of imported raw materials and artefacts is another novelty: carboniferous and cretaceous flint, particular slates and metatuffites as well as amber – and objects made of them. These materials and artefacts were transported in fairly large quantities and over distances of several hundreds, in some cases well over a thousand kilometres. Some artefact groups like wood-chopping tools of Russian-Karelian type or Baltic amber ornaments even indicate some sort of mass- or specialised production from the 4thmillennium calBCE onwards. The new exotic raw materials include also metal. The use of native copper started soon after 4000 calBCE in the Lake Onega region, from where it was also exported to Finland.

- Further, the formation or re-aligning of these contact or social networks and the changes they reflect or cause in the society are essential to the whole development

discussed here. As mentioned above, the old 'borders', which derive from the Mesolithic remained visible after the adoption of pottery in the research area. However, the strong Neolithic impulse around 4000 calBCE, which can, for example, be mirrored against the distribution of Typical Comb Ware, transcends these borders and shows a significant change in the prevailing networks. After several centuries this situation was changing again.

Recent aDNA studies have resolved the long-lasting debate between the migrationists and diffusionists about the origins of Corded Ware/Battle Axe Culture in the Eastern Baltic[23]. It has been shown that these populations migrated to the region from the area of Yamnaya culture at the Russian steppe[2,24]. This took place during the first centuries of the 3rd millennium BCE. This migration also brought about the onset of food production as a regular form of substinence in all the present Baltic States[7,25]. However, the new way of life – agriculture – did not mean an end to the hunter-gatherer populations. These existed parallel in the Eastern Baltic region for a while[23,25]. Differently from the hunter-gatherer sites the occupation layers of Corded Ware sites were rather thin[9,23]. Moreover, Corded Ware people preferred to settle farther from the shores of water-bodies, neither were their habitation sites known from the coastal areas[23]. The culture is mostly defined by the abundant grave finds – inhumations in flexed limbs and a specific set of grave goods comprising of battle axes and pottery –, and stray finds of boat shaped axes.

The beginning of Bronze Age in the Eastern Baltic is defined by the occurrence of the first bronze items, changes in the material culture in general, and the spread of bronze work. The earliest indications on the local bronze work in the region derive from Latvia and Lithuania around 2000 BCE[4]. The earliest bronze items show that Eastern Baltic tribes had contacts with different populations reaching from Scandinavia[26] to Russia[4]. Early Bronze Age has been seen as a transition period from Late Neolithic to the Late Bronze Age[4]. Despite the fact that many areas inhabited tensely during the Late Neolithic were abandoned and areas more appropriate for land cultivation were adopted during the Early Bronze Age, many previously known sites still remained settled; also stone tools still played a major role in the everyday life. Differently from Estonia EBA cemeteries are known both from Latvia (earth graves and barrows) and Lithuania (barrows) [4].

The classical characteristics of Eastern Baltic Bronze Age become visible during the LBA. Some of the most significant antiquities from the LBA are the fortified settlements that are known from all three Baltic States (e.g. Iru, Asva, Kivutkalns, Vinakalns, Narkunai, Nevieriškes). Several families resided in these sites, it has been estimated that the typical community ranged between 30 to 50 members[4]. In addition to the EBA barrows known from Latvia and Lithuania there are also stone graves with above-ground constructions known from Estonia. These circular stone-cist graves are the most studied objects from the given period[4]. Also boat graves and earth graves are known from this period. For the first time in the Eastern Baltic prehistory we have marks on ancient field-systems (clearance cairn filed, block-shaped fields: Baltic and

Celtic fields) that are well studied in Estonia[4]. These remarkable changes in material culture signify changes in the social structure and culture of the society. By the end of the period Eastern Baltic populations relied on economy fully focused on agriculture. These farming societies were characterized by stratified social structure developed ownership rights, rich material culture, and specific religious beliefs[4].

Kõnnu, Estonia

Kõnnu is located on the present island of Saaremaa at the Kõnnu village (Pihtla parish). During its habitation it was a separate island in Litorina Sea a couple of kilometres south-east from the larger island of Saaremaa[27–31]; according to analyses of the faunal material it has been argued to be a camp for seal hunting[13]. The settlement and burial site was discovered during gravel mining in the spring of 1977. The archaeological investigations lead by Lembit Jaanits took place in 1977 and 1978 (as the gravel mining continued documentation of the finds were carried out in 1979–1986). Most of the find material consisted of small quartz artefacts; amongst flint artefacts only scrapers were distinguished, also remarkable number of tooth pendants and various types of ceramics were found[27].

The site is from Late Mesolithic and Early Neolithic, and is attributed to Kunda, Narva and Combed Ware Culture[25,27]. The skeletal remains of four individuals were found in graves I, II, and III, whereas grave II contained the remains of two individuals[27]. In addition to these single human bones were gathered from non-grave archaeological contexts[25].

The biological sex and age at death estimations, and radiocarbon dates taken from Tõrv[25].

- Kõnnu I, juvenile, (KIA-49481, 6297±29 BP, 5320–5210 calBCE)
- Kõnnu IIa (prev. II), older child, (UBA-25997, 6222±33BP, 5310–5060 calBCE).
- Kõnnu IIb (prev. IIa), younger child. No direct radiocarbon dating.

Veibri quadruple burial, Estonia

The burial site is situated in south-eastern Estonia on a flood plain on the northern shore of the Suur Emajõgi River in the village of Veibri. The site was first discovered in 1997 and identified as a Corded Ware Culture and Medieval settlement site[32]; in 2003 bones exposed above the ground were detected. During the excavations (lead by Kristiina Johanson, Mari Lõhmus (Tõrv) and Tõnno Jonuks) of human skeletal remains a quadruple grave was unearthed. The archaeothanatological analyses revealed that this is a multiple burial[25], thus the deceased were buried simultaneously[25,33]; no grave goods were found despite Narva-type pottery sherd close to skeleton III[34].

The quadruple grave (TÜ 1424) is dated to the Late Mesolithic/Early Neolithic [25,34], biological sex and age at death estimation from Allmäe[33]:

- Veibri I/I child 12, (Ua-43124, 5580±39, 4490–4340 calBCE); (UBA-2735, 55791±43, 4770–4530 calBCE).
- Veibri I/II female 25–32 yrs, (Hela-1331, 6090±45, 5210–4850 calBCE).
- Veibri I/III child 5 yrs, (KIA-48842, 5841±29, 4790–4610 calBCE).
- Veibri I/IV child 4 yrs , (KIA-48843, 5940±22, 4900–4720 calBCE).

Kivisaare, Estonia

Kivisaare settlement and burial site is located in the southern part of Estonia in Lalsi village, approximately 1.5 km from Põltsamaa River and 6 km from present shore of Lake Võrtsjärv[34]. Here, the human skeletal remains have been found occasionally since 1882 during gravel mining. The around 30 individuals found there makes it one of the largest burial grounds in the present-day Estonia[25]. Several archaeological excavations on site have been conducted by different archaeologists since 1910 confirming human activity from Mesolithic to Bronze Age[12,35–40].

Kivisaare burials III, IIIa and IV were unearthed in 1960s (AI 4379)[41], IIIa burial was distinguished as a separate individual and labelled by Tõrv during osteological analysis[25]. The burials under study are attributed to Narva Culture and Combed Ware Culture[31]; Narva type pottery was found near Kivisaare IV burial[34]. Radiocarbon dates of burials from Kriiska[34] and Tõrv[25], biological sex and age at death estimations from Tõrv[25].

- Kivisaare III, male? adult (UBA-25993, 5796±37BP, 4730–4540 calBCE)
- Kivisaare IIIa, adult. No direct radiocarbon dating.
- Kivisaare IV, older child (Poz-10840, 5450±40BP, 4370–4230 calBC; KIA-50905, 5705±35BP, 4680–4450 calBCE)
-

Naakamäe, Estonia

Naakamäe is a settlement and burial site in south-western part of Saaremaa island, and is attributed to the Typical and Late Combed Ware Culture[12,18]. The settlement site was excavated by Lembit Jaanits during 1958–1962; one burial was found at the edge of settlement area. The body was buried on its back with upper limbs slightly abducted at the shoulder, the latter being projected upwards, arms medially rotated, and forearms flexed at the elbow and pronated, the hands being slightly flexed on the wrist and rested in front of the abdomen; the lower extremities were both extended from the hip and knee[25]. Beneath the skull ochre was found, and a bone awl (AI 4022) was placed next to left body side[12].

The biological sex and age at death estimation from Karin Mark's personal archive (TLU AI, F18)[42], and radiocarbon date from Lõugas et al. (1996)[43].

- Naakamäe I, female adult (Ua-4822; 4125±85, 2890–2480 calBCE)

Kõljala, Estonia

Kõljala is a burial site in southern part of Saaremaa island. Three burials were unearthed at Kõljala by Richard Hausmann in 1901 (AI K35)[44]. Tooth pendants and slate ring ornaments made of metatuff were found with one skeleton. Based on the latter find the graves were dated to the second half of the 3rd millennium[12], and attributed to the Combed Ware Culture[25].

The biological sex and age at death estimations, and radiocarbon dates after Tõrv[25]:

- Kõljala I, male? adult, (UBA-27363; 5180±44 BP, 4230–3810 calBCE)
- Kõljala III, male? adult, (UBA-25996; 4914±32 BP, 3770–3640 calBCE)

Tamula, Estonia

Tamula I settlement and burial site is located in southeastern Estonia on the north-eastern shore of Lake Tamula on a shallow cape Roosisaare, where Võhandu River joins the lake. The water level in Lake Tamula was lower during Stone Age than today therefore the cultural layer has been grown on the peat and sandmixed peat. Later the water level arose and the site was covered by lake[12].

Tamula I settlement and burial site was discovered in 1938, and has been archaeologically investigated during several decades: by Richard Indreko (1942–1943, AI 3932, burials I–III), by Harri Moora (1946, AI 3960, burials IV–VII) and by Lembit Jaanits (1955–1956, 1961, 1968, 1988–1989; AI 4118, burials VIII–XXV). The settlement and burial site is attributed to Combed Ware and Corded Ware Culture[12]. Tamula is outstanding of its amber artefacts, 191 finds have been recorded there[46], and of bone artefacts, including tooth pendants[12].

The skeletal remains of eight individuals were included to the study. The biological sex and age death estimations are given after Allmäe[47]; burial descriptions after Lohmus[48], Ots[46], and Tõrv[25] the radiocarbon dates from Lõugas et al.[43], Kriiska et al.[34], Mannermaa[43], and Tõrv[25].

- Tamula I, female 25–35 yrs, (Poz-15645 4680±40BP, 3630–3360 calBCE), buried on back, arms flexed from the elbow, with lower limbs flexed from the hip and the knee; grave goods present.
- Tamula III, male 25–35 yrs, (Poz-10826 4940±40BP, 3800–3640 calBCE), buried on back, arms and forearms flexed in front of the body and lower limbs flexed from the hip and knee, grave goods present.
- Tamula VI

- Tamula VII, child 8 yrs, (Hela-1335 5760±45BP, 4720–4500 calBCE), buried on back with both upper and lower extremities in extension; large bird bones and a stone; abundant grave goods present.
- Tamula VIII, female 18–25 yrs, (Hela-1336 5370±45BP, 4335–4055BC), buried on back with both upper and lower limbs in extension; wooden pole and branches; abundant grave goods present.
- Tamula IX male? 25-35 yrs.
- Tamula X, female 18–21 yrs, (Ua-4828 5310±85, 4330–3970 calBCE; UBA-27362 4902±52BP, 3890–3530 calBCE), buried on back, with both upper and lower limbs in extension; wooden branches; grave goods present.
- Tamula XI male? 18-25 yrs.
- Tamula XII child 2 yrs +-8 m.
- Tamula XIII child 2-3 yrs.
- Tamula XV, child ~1,5 yrs, buried on back, due to poor preservation the initial body position cannot be reconstructed entirely; grave goods present. No direct radiocarbon date.
- Tamula XVII, female 35–45 yrs, buried on back, with both upper and lower limbs in extension; grave goods present. No direct radiocarbon date.
- Tamula XVIII, female 25–35 yrs, buried on back, with upper limbs extended, lower limbs slightly flexed at knee; no grave goods (UBA-27359; 4696±39BP, 3640–3360 calBCE)
- Tamula XIX male 20–30 yrs.
- Tamula XX, male 25–35 yrs, buried on back, with both upper and lower limbs in extension; wooden branches; no grave goods. No direct radiocarbon date.
- Tamula XXI male 25–35 yrs.
- Tamula XXII, male? 25–35 yrs, (Ua-43123 4830±39BP 3700–3520 calBCE), buried on back with lower limbs hyper-flexed from the knee, placed behind the thighs; grave padded and covered with bark; no grave goods.
- Tamula XXIII male? adult.
- Tamula XXIV child, 2 yrs +-8 m.
- Tamula XXV adolescent.

Ardu, Estonia

Ardu cemetery is situated in Northern Estonia in the village of Ardu, in the area of the upper reaches of the Pirita River, 100 meters from the river, on a gravel hill rising above the surrounding river plain[12,34]. The site was discovered during gravel mining. Two burials – Ardu I burial (AI 2745) in 1931 and Ardu II burial (AI 3499) in 1936 –

were uncovered, both individuals buried in flexed position lying on left lateral side[49,50]. According to grave goods, for example Karlova-type battle-axe, bone adzes, flint adze, flint knives, bone awls, bone buttons, Corded Ware shreds etc., the burials are attributed to the Corded Ware Culture[12].

The biological sex and age at death estimations from Karin Mark's personal archive (TLU AI, F18)[42]; radiocarbon date of Ardu II skeleton (Poz-10824, 4110±40BP 2860–2580 calBCE 2880–2500 calBCE) from Kriiska et al.[34] and Lõugas et al.[43].

- Ardu I, male adult. No direct radiocarbon dating.

<u>Sope, Estonia</u>

Sope burial site is situated at Soonurme village in North-Eastern Estonia, 4 km east of Purtse and 2 km south of Jabara village, on the lands of Metsavälja farmstead, on a small sandy knoll on the bank of the Sope Stream[12].

Human skeletal remains and boat-shaped axes have been found since 1880s at Sope. The first proper archaeological excavations at Sope were conducted by Harri Moora [51], who uncovered strongly flexed female skeleton (Sope I; AI 2607). In 1933 Richard Indreko uncovered second female skeleton (Sope II; AI 3175) with legs flexed at knees, lying on her right body side, right hand beneath her head and left hand lying in front of the the body[52]. Both graves contained grave goods: shell of freshwater pearl mussels, bone awls, ceramic vessel, and are attributed to the Corded Ware Culture[12,34].

The biological sex and age at death are given after Karin Mark's personal archive (TLU AI, F18)[42]; radiocarbon dates of from Kriiska et al.[34] and Lõugas et al.[43] and Rasmussen et al.[53].

- Sope I, female adult, (UBA-29064; 3969±32, 2575–2349 calBCE).
- Sope II female adult, (Poz-10827; 4090±35BP, 2870–2490 calBCE)

<u>Kunila, Estonia</u>

Kunila burial site is situated in Central Estonia, 4 km south-west of Puurmani on the western side of a small drumlin on Jaaniantsu Hill. The burial site was discovered in 1938, when stone axe and loose human bones were found during gravel mining. During the archaeological excavations in 1948 skeletal remains of two individuals were uncovered (AI 3723)[54]. Kunila burials had grave goods: in connection to Kunila I a stone adze and a battle-axe were found; in connection to Kunila II burial an adze of white flint and a grinding stone were obtained. The burials are attributed to Corded Ware Culture[12].

The biological sex and age at death are given after Karin Mark's personal archive (TLU AI, F18)[40]; radiocarbon date from Kriiska et al.[34] and Lõugas et al.[43].

- Kunila I, male adult. No direct radiocarbon date.

- Kunila II, male adult (Poz-10825;3960±40BP, 2580–2340 calBCE)

Valma, Estonia

Valma is located in Central Estonia on the north-western shore of Lake Võrtsjärv, at the village Valma. The site was discovered in 1948, archaeological excavations lead by Lembit Jaanits were conducted in 1949, 1950, and 1953–1955. Although the majority of the settlement area was studied no other constructions than c. 10 hearths were found[55]. These allowed concluding that three to five contemporaneous dwellings must have been part of the settlement[18]. Also, six hearths with stone packing were found on the upper horizon of the cultural layer; these could either belong to the Typical Combed Ware or the Corded Ware Culture[18] or even to the Late Iron Age. The majority of the find material is Typical Combed Ware sherds[18,55], but shards of Late Combed Ware and Corded Ware were also present. Also small stone tools from local flint were found[18,55], and various kinds of adzes[18,55].

From the periphery of the settlement area (NW part) two individuals – an adult male (III) and a young female (II) – were found[25]. These were adorned with grave goods[18,55]. Another grave with the remains of a child (I) in a depth of 115 cm from the topsoil was found on the SE part of the settlement. In this case only the skull and some other bones[12,18,55] were detected.

Due to the poor collagen preservation none of the three graves are dated[25]. Grave descriptions given after Tõrv[25].

- Valma I, a child; partially preserved; without grave goods. No direct radiocarbon date.
- Valma II, an adult male; buried on back with both upper and lower limbs in extension; grave goods present. Probable collective burial, placed into the same grave with individual no. III. No direct radiocarbon date.
- Valma III, a young woman; buried on back with both upper and lower limbs in extension; grave goods present. Probable collective burial, placed into the same grave with individual no. II. No direct radiocarbon date.

Kääpa, Estonia

Kääpa is a settlement site located on the left bank of the Võhandu River, c. 5–10 km north-east of the Tamula settlement and burial site. The site was discovered in 1958 during the construction work of a bridge, and excavated in 1959–1962 and 1974 by Jaanits[56]. Similar to Tamula and Akali, the cultural layer at Kääpa is covered with peat, which indicates that during the habitation of the site, the water level at Võhandu River was lower than it is today. The find material is plentiful, being mostly represented by Narva Ware, but also Typical Combed Ware sherds have been found[56]. During the excavations in 1962, single human bones and cranial fragments were found from Kääpa.

- Kääpa I, an almost complete cranium with maxillary teeth of an adult individual are stored in the Archaeological Research Collections at the Tallinn University (AI 4245). No direct radiocarbon date.

Narva Joaorg, Estonia

Narva Joaorg is located near the present town of Narva on the eastern border of Estonia of the western bank of the Narva River. The site was discovered in 1953 and in 1954 and 1957 Jaanits carried out preliminary surveys that were followed by extensive excavations in 1960 and 1962–1964. Narva Joaorg is a multi-layered and multi-cultural site consisting of three pre-ceramic Mesolithic layers and a Mesolithic/Early Neolithic layer with both Narva Ware and Typical Combed Ware present[12,18,57,58]. The oldest settlement layer was situated directly above the limestone bedrock. The radiocarbon dates from the Mesolithic layers date the site to c. 6600–4200 cal. BC, being the earliest known human occupation in the Narva-Luga region[59].

The graves (AI 4264) comprise of loose, i.e. not fully articulated, human bones. No direct radiocarbon dates are available from human bone due to poor collagen preservation[25]:

- Narva Joaorg I, probable male, adolescent, in the I Mesolithic layer, no grave goods. No direct radiocarbon date.
- Narva Joaorg II, an adult, in the II Mesolithic layer, no grave goods. No direct radiocarbon date.
- Narva Joaorg IV, an adult, in the III Mesolithic layer, grave goods. No direct radiocarbon date.

Biržai, Lithuania

The single burial site is located within the limits of the Biržai town in the northern part of Lithuania. Grave was originally placed at the edge of a steep and clayey bank (elevation up to 53 m a. s. l.) of the river Apaščia, some few hundred meters south from where it meets one of its tributaries – the river Agluona. In the 16th c. the dam was built on the confluence of above-mentioned rivers, creating the artificial lake Širvėna. The placement of the burial site in the landscape is very similar to Benaičiai case as there are valleys of the rivers Apaščia to the east and Agluona to the west as well as a steep slope descending to what used to be the wet grasslands that are flooded at the present.

The grave was partially destroyed during the construction works in 2013 and then rescue excavations took place in 2014 uncovering the single grave dating to the Late Neolithic period[60,171].

- Biržai 1, 30-35 year old male (Poz-64678; 3955 ± 30 BP, 2570–2350 calBCE)

Donkalnis settlement and burial site

The site comprises of a settlements dating to the Mesolithic, Late Neolithic and Early to Middle Bronze age and burials dated to a Mesolithic and Neolithic periods. It is located in the central north-western part of Lithuania. It is situated on a glacial kame formation (elevation up to 156 m a. s. l.) towering above the marshy northern part of the lake Biržulis where the rivulet Druja flows into it. In some stages of the Stone Age the kame may have been an island[61–63].

After the first melioration of the lake in the 1930's the site became accessible on foot and local residents started exploiting it for gravel quarrying. The site caught archaeologists' attention only in 1979 when a local child told them about human bones being constantly uncovered during gravel extraction. Site was excavated in 1981-1983) resulting in the discovery of 7 undisturbed inhumation graves among numerous pottery fragments, flint artefacts and pits. Graves 4 and 5[8,171] were radiocarbon dated to the Mesolithic, while grave 6 and 7 were directly dated to the Middle Neolithic[171,172], and grave 1 assigned to the same period based on associated artefacts. Minimal number of individuals (MIN) determined from the bones collected in the vicinity of the destroyed part of the kame shows that graves of at least 6 more individuals were destroyed during quarrying[62].

- Donkalnis 1, ~20 year old female, no direct dating.
- Donkalnis 4, 50-55 year old male (OxA-5924; 6995 ± 65 BP, 6000–5740 calBCE)
- Donkalnis 5, child (Poz-61589; 7140 ± 40 BP 6075–5920 calBCE)
- Donkalnis 6, 35-40 year old female (Poz-61574; 5770 ± 40 BP, 4720–4530 calBCE)
- Donkalnis 7, ~50 yeaor old male (Poz-61576; 6220 ± 90, 5460–4940 calBCE)

Gyvakarai burial site

The burial site is located in the northern part of Lithuania on the steep gravelly bank (elevation up to 79 m a. s. l.) of the rivulet Žvikė, 500 m to the south from where, in the wet grassland valley, it meets the main stem river Pyvesa.

The site was discovered in 2000 when local residents started digging for gravel in the central part of the gravelly bank. The same year rescue excavations were conducted in the surrounding area of the highly disturbed grave resulting in discovery of a single grave C14 dated to the Late Neolithic[64,171].

- Gyvakarai 1, 35-40 year old male (Poz-61584, 4030 ± 30 BP, 2620–2470 calBCE)

Kretuonas settlement and burial site

The Neolithic – Bronze Age settlement and the Neolithic burial site is located in the eastern part of Lithuania. It is situated on a small sandy height (elevation up to 147 m a. s. l.) edged between the lake Kretuonas to the west and marshy remains of the

nameless grown up lake to the east[65]. Surroundings of the lake Kretuonas are rich in a variety of settlements and burial places dating from the Stone to the Early Middle ages.

The site was discovered in 1978 when the area was being planted with forest. The same year archaeological excavations were started and continued for thirteen years (1978-1980, 1982-1985, 1991, 1994-1997, 1999, 2001). In 1980, six undisturbed graves dating to the Early – Middle Neolithic periods were uncovered in the low-lying and intermittently flooded periphery of dwelling area[8,171]. Uncovered graves themselves and their placement in the surrounding landscape arguably show signs of unusual burials.

- Kretuonas 1, 20-25 year old female (OxA-5935, 5350 ± 130 BP, 4460–3820 calBCE)
- Kretuonas 2, 14-16 year old juvenile, no direct dating.
- Kretuonas 3, 50-55 year old male (OxA-5926, 5580 ± 65 BP, 4550–4330 calBCE)
- Kretuonas 4, >55 year old female, no direct dating.
- Kretuonas 5, 25-30 year old male (Poz-64677, 5540 ± 35 BP, 4450–4340 calBCE)
- Kretuonas 6, infant, no direct dating.

Spiginas settlement and burial site

The Neolithic – Bronze Age settlement and the Mesolithic – Neolithic burial site is located in the central north-western part of Lithuania. The settlement is situated on a terrace (elevation up to 156 m a. s. l.) in the centre of which is a glacial kame formation (elevation up to 163 m a. s. l.) with a burial site on top of it. In some stages of the Stone Age this terrace and kame may have been an island in what is now a marshy southern part of the lake Biržulis[61,63].

This site is different from the others because it was discovered by professional archaeologists from the very beginning, which led to two years of excavations (1985 – 1986). During these excavations 4 graves were discovered and C14 dated to the Middle Mesolithic, Middle Neolithic and Late Neolithic periods[8,62,171,172].

- Spiginas 1, 35-45 year old male (Poz-61572, 5470 ± 40 BP, 4440–4240 calBCE)
- Spiginas 2, 50-55 year old male (Poz-61573, 3580 ± 60 BP, 2130–1750 calBCE)
- Spiginas 3, female (OxA-5925, 7780±65BP, 6400–6240 calBCE)
- Spiginas 4, 30-35 year old female (Gin-5571, 7470 ± 60 BP, 6440–6230 calBCE)

Plinkaigalis burial site

The burial site is located in the plains of central Lithuania on the eastern bank of the river Šušvė on the outskirts of the Plinkaigalis village, approximately 400 m south-

east of an Iron age hill fort and settlement[66]. It is a relatively plane area, which is nevertheless distinguished from the surroundings by a low (up to 2 m height) moraine hill of sand and gravel (elevation up to 94 m a. s. l.).

The burial site was discovered in 1975 when local residents started digging for gravel in the western part of the hill. The same year site was granted a legal protection with archaeological excavations carried out for eight straight years in a row (1977-1984). During the eight years of fieldwork a total of 373 graves (364 inhumation and 9 cremation graves) with all but two of them dating to 3rd to 8th c. AD were uncovered[66]. The two exceptional graves (no. 241, 242) were uncovered in the northern part of the burial site and C14 dated to the Late Neolithic[67].

- Plinkaigalis 241, 50-55 year old female (OxA-5928, 4030 ± 55 BP, 2860–2410 calBCE)
- Plinkaigalis 242, >40 year old female (OxA-5936, 4280 ± 75 BP, 3260–2630 calBCE)

Turlojiškė archaeological complex, Lithuania

The Turlojiškė archaeological complex is a group of settlement, burial, sacrificial, and other archaeological sites in a large, peaty area on the right bank of the river Kirsna in south-western part of Lithuania. The relief of this area is almost level, slightly undulating in places, with elevated areas in the northern and southern parts. The river Kirsna is in the western part of the area, the small Turlojiškė forest in the east, the grove Gojus located in the north on the grounds of the Jakimavičius family farmstead, and level cultivated fields in the south[68]. The first written record about archaeological finds from the area reached us from 1930[69], in 1931 another paper was written on a human skull called „Kirsna man“[70], which later turned out to be a skull of 35-40 year old male[71].

A more precise location of the find spot of the aforementioned finds was not known until field surveys and excavations were conducted from 1995 to 2003. During the excavations 6 additional male graves were discovered and some of them were radiocarbon dated to Bronze age[8,68,171].

- Turlojiske 3, 25-30 year old male (Vs-1188, 2736±60 BP, 1010-800 calBCE)
- Turlojiske 4, 20-25 year old male (Ua-16681, 2590±75 BP, 908-485 calBCE)
- Turlojiske 1999.5, 20-25 year old male.
- Turlojiske 1999.6, 20-25 year old male.
- Turlojiske1996.1, 35-45 year old male (Poz-66904, 2730 ± 30 BP, 930–810 calBCE)
- Turlojiske 1932, 20-30 year old male („Kirsna man“) (OxA-5931, 2895 ± 55 BP, 1230–920 calBCE)

- Turlojiske 1948, 25-35 year old male.

Kivutkalns, Latvia

The Ķivutkalns hill-fort and cemetery was located on the island of Dole, on the lower course of the River Daugava, on a sandy spit of land formed by the former shore of the Daugava and the bed of a former river channel that is nowadays hard to distinguish. The Ķivutkalns site was totally excavated under the direction of Jānis Graudonis in 1966 and 1967 in connection with the building of the Riga Hydroelectric Plant.

The site has been considered as the largest Late Bronze Age bronze-working centre in Latvia. One third of the archaeological artefacts found at Kivutkalns hill-fort in the lower Daugava river is related to bronze working[72]. The Ķivutkalns was a double monument: according to archaeological evidence, a fortified residential site had been established directly on top of what had originally been a burial site. This in itself is an unusual, even unique case: the establishment of a hill-fort on top of a prehistoric cemetery. Based on ^{14}C dating and artefactual dating, the hill-fort was placed in the 1st millennium BC[73]. Radiocarbon datings of bone samples (by the Finnish Museum of Natural History, 2008 and 2011) indicated 2543 to 2576 years BP for animal bones, found in the hillfort, and 2475 to 2555 BP for human bones, found in the cemetery.

Combining the new animal bone data to old radiocarbon dates of charcoal provides supporting evidence for archaeological consensus date of the hill-fort usage during the first millennium BC. Five human bone collagen ^{14}C datings are surprisingly young and suggest that periods for cemetery and hill-fort usage were overlapping[74].

The cultural layer, with a thickness of 1.6–3 m protected the cemetery from the harmful effects of the atmosphere and precipitation and in combination with the encasing of the burials in oak log it resulted in an exeptionnaly good preservation of the skeletons. All the burials in the cemetery were preserved (247 inhumations and 21 cremations)[75].

For this work, all the studied individuals were radiocarbon dated and the results are given below (see also Table 1). Generally, the dates are consistent with the earlier measurements. Full discussion on the dates incorporated with dietary isotopic signals and potential reservoir effects is published elsewhere[76].

- Kivutkalns 19. Male aged 40-50 (Hela-3746, 2403 ± 24 BP, 730-400 calBCE), on the back in an oak log. A bone needle on the breast. 14 stones arranged around burial, white sand strewn below it.

- Kivutkalns 25. Male aged 35-40 (Hela-3738, 2545 ± 30 BP, 800-545 calBCE), on the back in an oak log. 4 stones arranged around burial, white sand strewn below it.

- Kivutkalns 207. Female aged 40-50 (Hela-3743, 2511 ± 30 BP, 790-535 calBCE). in an oak log. White sand strewn below burial. A bone needle on the back.
- Kivutkalns 194. Male aged 30-40 (Hela-3737, 2298 ± 28 BP, 405-230 calBCE), on the back in an oak log. A bone needle on the breast.
- Kivutkalns 209. Male aged 25-33 (Hela-3741 and 3742, 2497 ± 30 and 2556 ± 30 BP, 785-515 and 805-550 calBCE) in an oak log. Stones arranged at the head part of burial. White sand strewn below burial. Both mandible (Hela-3741) and maxilla (Hela-3742) were dated.
- Kivutkalns 215. Disturbed. Female aged 18-22 (Hela-3745, 2462 ± 27 BP, 760-425 calBCE), without any artefacts.
- Kivutkalns 42. Female aged 35-42 (Hela-3740, 2573 ± 30 BP, 810-560 calBCE), possibly in a linden coffin. No artefacts.
- Kivutkalns 222. Male of big stature, aged 60-70 (Hela-3736, 2423 ± 26 BP, 745-400 calBCE), on the back in an oak log. A fragment of bone needle at the left side of the breast. A stone at the head of burial. White sand strewn below burial.
- Kivutkalns 153. Male aged 45-55 (Hela-3744, 2542 ± 29 BP, 800-545 calBCE), without artefacts.
- Kivutkalns 164. Aged 18-25 (Hela-3739, 2385 ± 30 BP, 730-390 calBCE), without artefacts.

Scandinavia

Archaeological finds show a human presence in Denmark and the south Swedish province of Skåne during the Lateglacial, when most of the Scandinavian peninsula still was covered by glaciers[77–80]. The ensuing Preboreal period saw a rapid colonization over large tracts of lands, with settlements established all along the coasts of Norway and West Sweden[81–85], and also some sites known from Eastern and Northern Sweden as well as Northern Finland[86–88]. The Early Mesolithic archaeological assemblages differ between the South-West and the North-East, suggesting that groups with contrasting technological traditions entered the Scandinavian peninsula from different directions: One northern route through Finland that brought the post-Swiderian tradition, and a southern route through Denmark that brought the post-Ahrensburgian tradition[84,89,90]. It is also possible that humans crossed the Baltic by boat or on the sea ice of winter. Regional differences in material culture and technological traditions during the subsequent millennia of the Mesolithic[90–93] may partly be related to patterns that were already established during the colonization process, but may also be a result of later spread of traditions through social networks and/or resettling of groups of hunter-gatherers.

Around 4000 calBCE agriculture was introduced into South Scandinavia, along with a characteristic set of material culture known as the Funnel Beaker Culture (FBC or in

the following TRB after the German term *Trichterbecherkultur*)[94–99]. There are a few examples of domesticates that pre-date this event, in the form of carbonised cereal grains, imprints of cereal grains in Mesolithic pottery and bones from domestic animals[98–101], but it is only after 4000 calBCE that evidence of farming and cattle rearing becomes abundant in Denmark and South Sweden. Cattle was not only a source for meat, but was also used for dairy production[99,103–105]. Material culture that can be considered part of the Funnel Beaker tradition also appear in South Norway, but here evidence of agriculture remain elusive or at best sporadic[106-111]. In the region of Denmark and South Sweden where agriculture do occur, it was practised along side hunting, gathering and fishing[94,95,99,103].

In Southernmost Sweden, Denmark and adjacent Northern Germany pottery was produced locally already during the Mesolithic Ertebølle phase. The introduction of the TRB pottery tradition meant a change in technology and design that can be viewed as sudden or gradual depending on perspective[95,98,100,112,113]. In Central Sweden and Southern Norway TRB pottery was the first ceramic tradition to appear, and thus clearly have an origin outside of these regions[97,109]. The lithic industries tend to show a degree of continuity between the Late Mesolithic and Early Neolithic in most areas[96,110,114]. From a regional perspective, it is possible to distinguish TRB regions with specific designs and traditions that more or less mirror the geographic extent of Late Mesolithic traditions. This is also true on a pan-regional scale, where the Northern limit of the Scandinavian Funnel Beaker Culture coincide with the Northern limit of Late Mesolithic traditions[96,115].

The nature of the transition to the Neolithic in Scandinavia has been debated since the birth of Scandinavian archaeology, and the change has variously been put down to migration or internal development[116]. During the last couple of decades two basic models has dominated the Scandinavian archaeology. One model sees the introduction of agriculture as a consequence of interaction, gift giving and intermarriage through social networks that connected foragers and farmers[95,101,115]. The other model envisions agriculture as brought by migrating farmers seeking new lands[98,117,118]. The former model put emphasis on the *continuity* between the Late Mesolithic and the Early Neolithic in terms of lithic technology and complex subsistence practices like seal hunting. The latter model put emphasis on elements that show *discontinuity* like new designs of material culture and the introduction of complex new subsistence practices like cultivation and dairy farming.

At the transition between the Early and Middle Neolithic, c. 3300 calBCE, several changes can be seen in the archaeological record of Sweden. In Skåne and Western Central Sweden a large number of megaliths (dolmens, passage graves) were erected, and a new set of pottery and stone tools of new designs were introduced[102,119,120]. This material is known as the Middle Neolithic Funnel Beaker Culture. The change seems to be accompanied by a larger reliance on agriculture[121,122].

The development was quite different in Eastern Central Sweden, where the settlement pattern shifted to the coast[123]. All known interior TRB settlements of an agricultural

character were abandoned, and coastal sites previously used as seasonal camps for fishing and sealing were turned into more or less permanent settlements[96,123,124]. While there are traces of small scale agriculture at several of these coastal localities (eg. carbonised cereal seeds)[125,126], the bulk of the subsistence economy seem to have shifted towards marine resources like fish and seal[123,125–129]. The economic change was accompanied by a change in material culture, particularly pottery design[123]. The archaeological assemblages from this phase are known as the Pitted Ware Culture (PWC). It should be emphasised that the change in material culture was gradual, and that the pottery of the first phase of the PWC retained the funnel-beaker shape, with changes mainly confined to the composition of the decoration, and the surface treatment of the vessels[123,124,132].

The archaeological terminology with monikers like “Early Neolithic Funnel Beaker Culture”, “Middle Neolithic Funnel Beaker Culture”, and “Pitted Ware Culture” seem to imply continuity between the Early and Middle Neolithic TRB, and discontinuity between the EN TRB and PWC. However, it is important to recognise that this terminology was caused by research historical happenstance during the early 20th century[125,132,133]. From the perspective of material culture, there is as much continuity between the EN TRB and the PWC, as between the Early and Middle Neolithic TRB.

The phase of the PWC that display a clear continuity with the preceding EN TRB, the Fagervik II stage, is primarily known from the former EN TRB areas of mainland and coastal archipelagos of Eastern Central Sweden[125,134]. The early PWC also appear in the regions immediately to the north of the EN TRB area, regions that were occupied by aceramic hunter-gatherers during the preceding phase (the latter groups are sometimes included under the term Slate Culture)[124,133,135]. This may be a territorial expansion of the EN TRB turning PWC, or local hunter-gatherers finally adopting the ceramic technology of their neighbours. An indication that the latter scenario may be right is the fact that there is a simultaneous transfer of technology going in the other direction: the use of the raw material slate for the production of polished arrow and spear points that become an important part of the PWC[124,136].

During the following phases (Fagervik III, IV etc) the Pitted Ware phenomen spread over a larger area[137], including the Baltic islands of Öland and Gotland (Sweden) and Åland (Finland). While the former two islands have traces of a TRB presence during the Early Neolithic, the Åland island had for millennia been settled by groups of hunter-gatherers of the Comb Ware tradition[91,138]. Stylistic borrowings from the Comb Ware tradition can be traced already in the EN TRB of Eastern Central Sweden, and the creation of the material culture of the PWC can be seen as the continuation of this process, with a blend of western and eastern ceramic traditions[123,124,133,139]. During the later phases of the PWC the funnel-beaker shape was abandoned and instead pots were designed with pointed bottoms, akin to East Baltic hunter-gatherer pottery.

From an archaeological point of view then, the Pitted Ware Culture could perhaps be seen as a mix of at least three components: The Early Neolithic Funnel Beaker

Culture of Eastern Central Sweden, the Slate Culture of Dalarna and southern Norrland, and the Comb Ware Culture of Åland. It is likely that such a process also had consequences for the genetic composition of the resulting population.

Around 2800 calBCE – at the transition between Middle Neolithic A and Middle Neolithic B according to the Scandinavian terminology – a new set of material culture appear in the archaeological record of South Sweden and Norway, referred to as the Swedish-Norwegian Battle Axe Culture[119,123]. This is a regional version of the pan-European Corded Ware Culture (CWC). The Swedish CWC is represented by graves and - more rarely - settlement sites throughout South and Central Sweden, with a northern limit at the 60th parallell North. In the south Swedish province Skåne there are also palisade structures known[140]. In many regions the archaeological remains of the CWC replace or follow on the preceding MN TRB. This has variously been interpreted as the result of internal development, limited population movements or full scale migrations[122,139,141,142]. Recent research has stressed the similarities between the early phase of the Swedish CWC and the CWC of Finland, the Baltic Countries and northern Poland[122].

In eastern South and Central Sweden the CWC appear alongside the Pitted Ware Culture[117,122]. While the latter have a clear association with the coast, due to the subsistence focus on fishing and sealing, the settlements and graves from the CWC are often found in the interior, without a direct association with water. The data on the subsistence of the CWC is sparse, but include domesticated animals, cereal crops and wild forest game[117,120,122,143].

The northernmost provinces in Sweden with settlements and graves of the traditional CWC are Uppland and Västmanland in eastern Central Sweden. Most of Northern Sweden remained the territory of hunter-gatherers, but there also appear a small number of sites along the coast that contain archaeological material associated with the CWC[144]. These sites are of two types, one of which have been interpreted as the result of direct long distance (1500 km) colonization by CWC groups originating in South Scandinavia, the other type show a larger degree of continuity with the local hunter-gatherer traditions, but with with additions of cultivated plants and stylistic or technological borrowings that suggest contact with the CWC tradition.

The former category is best exemplified with a series of sites in Västerbotten, eg. Bjurselet, a settlement where close to 200 flint axes have been found, despite being located some 1500 km north of the nearest flint sources of South Scandinavia[144–146]. The lithic reduction done at Bjurselet suggest that the knappers where schooled in the technological tradition of South Scandinavia[145]. The other category is examplified by the sites Hedningahällan in Hälsingland and Bjästamon in Ångermanland[148–150]. These settlements contain lithic industries typical of the local hunter-gatherers eg. tools of slate and quartz, but in the case of Hedningahällan also fragmented CWC battle-axes, osteological assemblages dominated by seal and fish, but also finds of carbonised cereal seeds. The pottery is of a regional character, although with some

stylistic traits reminiscent of Corded Ware[150]. The human crania from Ölsund, Forsa, Hälsingland discussed in the current paper has been found in the same region as the site Hedningahällan and date to the same phase[151].

Kanaljorden, Motala, Sweden

Kanaljorden, Motala, is a Mesolithic settlement and ritual complex in the town of Motala, Östergötland, Sweden[86,152]. Motala cradles the outlet of the big lake Vättern into the river Motala Ström, which in turn discharge into the Baltic Sea. The site Kanaljorden (Motala parish ancient monument 187) is close to both river (100 m) and lake Vättern (300 m), but is situated on the rim and bottom of a separate wetland basin that constituted a small isolated lake during the time in question. Today the area is a peat bog, and is also covered by recent landfill and development.

The human remains that has been subjected to DNA-analysis where found on a man made 12 x 14 m stone-packing that had been constructed on the bottom of the small lake during the Mesolithic. The stone-packing was completely submerged and covered by at least 0,5 m of water at the time of use. The ritual depositions include human bones, mostly skulls and fragments of skulls of adults (21 finds) but also some stray postcranial bones from adults (10) as well as the bones of one infant. The latter find include bones from all parts of the body. The initial sampling for DNA of the Kanaljorden material was done before to the full osteological analysis to avoid DNA contamination. Each isolated find of a crania/crania fragment with part of the maxilla preserved, was numbered as individuals 1-9 during the sampling process, while the bones of the infant was numbered individual 10. A fragmentary find that was assigned as 11 during sampling, was retracted before analysis as the bones turned out to be non-human (was first believed to be fragments of a child crania). A disarticulated mandible was designated individual 12.

The osteological analysis that were done later calculated the MNI to ten based on the number of occipital bones, nine adults and one infant. This match the preliminary approximation based on the number of maxillas, although based on different bone elements[153].

The DNA analysis of six of the crania and the mandible confirmed that each of these finds represents a unique individual[1]. Osteological observations and isotope analysis furthermore show that the mandible do not match any of the three remaining "individuals" from whom no DNA data are available[153,154]. Furthermore, considering the result of isotope analysis and overlap of bone elements, one fragmentary skull that lack a maxilla can be recognised as an additional individual, given the designation AA. In all this mean that a total of 12 individuals so far has been identified in the Kanaljorden assemblage (individuals 1-10, 12 and AA).

Two of the skulls were mounted on wooden stakes still embedded in the crania at the time of discovery, indicating that the skulls have been put on display prior to the deposition in to the lake. Beside human bones, the ritual depositions also include artefacts of antler, bone, wood and stone, animal carcasses/bones as well as nuts,

mushrooms and berries. The fauna assemblage is dominated by bones from wild boar and brown bear, but also include elk (moose), red deer, roe deer, badger, beaver, pond turtle and pike. The depositions of artefacts include smooth bone points, barbed bone points, a bone point with flint inserts, antler pick axes and scrapers/knives made of wild boar tusk.

Direct dates on 18 human bones range between 7013 ± 76 and 6677 ± 31 BP, disregarding one outlier (7212 ± 109) which could not be confirmed by a duplicate date (6773 ± 30). Dates on animal bones (N=13) and tools made of bone and antler (7) range between 6935 ± 47 and 6611 ± 30 BP. The human samples may be affected by a moderate reservoir effect[154]. The time range indicated by the series of dates correspond to the end of the Middle Mesolithic of Scandinavia.

- Motala 300, ind. 7, skull mounted on wooden stake, 30-50yrs. (Ua-42121, 7013 ± 76 BP, 6016-5739 calBCE)
- Motala 307, ind. AA, adult female, same as Motala 317 and 1913.
- Motala 309, ind. BB, male, >30yrs, poss. same as ind. 6 (Ua-51717, 6836 ± 32 BP, 5781-5650 calBCE)
- Motala 311, adult (Ua-51718, 6965 ± 31 BP, 5971-5751 calBCE)
- Motala 313, adult (Ua-51719, 6758 ± 32 BP, 5716-5626 calBCE)
- Motala 314, ind. BB, poss. same as ind. 6
- Motala 317, ind. AA, adult female, same as Motala 307 and 1913
- Motala 318, skull mounted on wooden stake, adult (Ua-51720, 6770 ± 31 BP, 5719-5631 calBCE)
- Motala 363, ind. 1, adult female (Ua-42116, 6701 ± 64 BP, 5721-5515 calBCE)
- Motala 1913, ind. AA, adult female, skull fragment found inside skull of ind. 1, same as Motala 307 and 317 (Ua-42645, 6735 ± 44 BP, 5722-5564 calBCE)
- Motala 4352, skull fragment with injury from a pointed object, adult >25 yrs (Ua-51721, 6896 ± 31 BP, 5844-5718 calBCE)

Kvärlöv Kalkkälla, Saxtorp, Sweden

The site Kvärlöv Kalkkälla (Saxtorp parish ancient monument 61), Skåne, Sweden is an offering-fen of the Funnel Beaker Culture (TRB), situated in the outskirts of the modern village Kvärlöv[155]. The fen is characterised as a spring mire, and is believed to have been rather shallow with varying water level. The material under consideration here date to the Scandinavian Early Neolithic (4000-3300 calBCE). During the Early Neolithic the spring mire was located on a gentle slope facing a narrow bay of the sea, some 50-100 m away, and 200 m from the mouth of the small river Kvärlövsån.

The Early Neolithic (EN) assemblage was found in a layer of sand mixed with fen peat. The find bearing EN layer has an extent of 350 m^2, which gives a rough estimate

of the extent of the wetland at this time[155,156]. The finds include 39 disarticulated human bones (0,7 kg). Three fragmentary crania are designated as numbered individuals 1, 2 and 3 in the osteological report, albeit with some reservation regarding the identification of Individual 3 in relation to Individual 2[155]. As the radiocarbon dates from these two finds are significantly different (Test statistic T 7.22, Xi2(.05) 3.84, degrees of freedom 1) they likely represent two different individuals. The remaining human bones may represent the same or additional individuals, seven of these have been included in the initial screening for aDNA.

Besides human bones, the spring mire also contained fragmented funnel-beaker pottery, knapped flint, hazelnut shells, raspberry seeds and animal bones. The fauna assemblage is dominated by domesticated species (cattle, sheep/goat and pig) but also include various wild animals as roe deer, red deer and seal. A dagger made of cattle bone has been ^{14}C-dated to the same period as the human remains (Ua-25501 4810±75), while bones from sheep/goat and pig are slightly younger and fall in the beginning of the Scandinavian Middle Neolithic (Ua-25499 4470±60, Ua-25500 4550±60)[151].

There are two Early Neolithic TRB settlement sites in the immediate vicinity, the site Saxtorp (Saxtorp parish ancient monument 26), just 50 m North of the spring mire, and the settlement site Kvärlöv (Saxtorp parish ancient monument 23) 350 m to the South-East. The Early Neolithic phase at the Saxtorp settlement is represented by one singled ailed house, 19 x 6 m, and associated features and finds. The find material include funnel-beaker pottery, cereal seeds, hazelnut shells and knapped flint. ^{14}C-dates from features in the house indicate that it was in use during the later phase of the Early Neolithic. The settlement site Kvärlöv date to the first half of the Early Neolithic, and contain an oval post built house, 7,5 x 4,5 m, as well as two graves (skeletons not preserved) and features interpreted as wells or watering pits. The finds include fragmented funnel-beaker pottery, knapped flint, teeth from cattle, cereal seeds and a quernstone[97,155,157,158].

- Saxtorp 389, adult <40 yrs.
- Saxtorp 462T, older adult.
- Saxtorp 4815.
- Saxtorp 316_5527_5817
- Saxtorp 5092, ind. 1, 20-30 year old female (Ua-9810, 4760±75 BP; Ua-9811, 4520±80 BP, mean 4648 ± 55 BP, 3631-3140 calBCE)
- Saxtorp 5094, ind. 2, adolescent or young adult female (Ua-9809, 4690 ± 75 BP, 3645-3196 calBCE)
- Saxtorp 5158T, adolescent (Ua-52673, 4686 ± 28 BP, 3625-3371 calBCE)
- Saxtorp 5164, ind. 3, adolescent (Ua-9808, 4975 ± 75 BP, 3945-3647 calBCE)
- Saxtorp 5477, ind.3, same as Saxtorp 5146.

- Saxtorp 5478, same individual as either 5092 or 5094.

Ölsund, Hälsingland, Sweden

The human cranium from Ölsund, Forsa parish, in Hälsingland is a stray find exposed by ditching of wetland forest[151]. The find-spot (Forsa parish ancient monument 416) is below the Stone Age sea level, the crania may be the remains of a drowned individual, or a person buried in the sea. Ölsund is located 20 km from the contemporary settlement site Hedningahällan, with an assemblage that show a mix of local hunter-gatherer tradition and CWC influences (cf. above). The crania belong to an individual between 28 and 45 years of age[159]. Compared to a modern skeleton, the features are robust, but less so than many males from Stone Age Scandinavia. There are some traits that point towards the identification of the cranium as female (*tubera frontalia* and *occipitalia*).

- Olsund, 28-45 y old adult (Ua-2138, 3890±80 BP, 2573-2140 calBCE)

Northwest Russia

The first indications of settlement in the region of Karelia in the northwest of Russia and southwest of Finland date back to the Mesolithic, with the earliest find of a fishing net, radiocarbon dated to 9310±120 BP[160].

This region is relevant to the peopling of Scandinavia after the retreat of the glacial ice sheets, as it lies on one of the proposed eastern routes of settlement[88], which has also been suggested for the introduction of Saami-like groups[161]. Odentometric analyses suggested continuity between the Mesolithic population of Yuzhnyy Oleni Ostrov and present-day Saami[162].

Yuzhnyy Oleni Ostrov, Karelia, Russia

The Mesolithic site Yuzhnyy Oleni' Ostrov (*Oleneostrovski' mogilnik* or *Deer Island cemetery*), Onega Lake, Karelia, Russian Federation (61°30'N 35°45'E) was first discovered in the 1920s during quarrying activities, which unfortunately resulted in the destruction of large parts of the graveyard. Archaeological examination and professional excavation of the site by Soviet archaeologists in the 1930s and the 1950s eventually led to the discovery of 177 burials[163], while the original size of the burial ground was estimated to have held up to 500 individuals[164]. The abundance and diversity of grave goods and various mortuary features render the Yuzhnyy Oleni Ostrov site exceptional among other Mesolithic sites in Europe. The site had first been identified as a Neolithic graveyard, but later reanalysis and radiocarbon dating revealed an age of around 7,700–7,300 uncalibrated years BP[165,166]. Skeletal remains and artifacts from this site are currently held at the Kunstkamera Museum, St Petersburg, Russia.

Co-analyzed with new data is the genome-wide data of UzOO-40 and UzOO-74 from

this site which has been reported in previous studies[2].

- UzOO77, female, 7,450-6,950 calBP, layer date based on associated individuals from the graveyard[165]

Popovo, Archangelsk, Russia

The Mesolithic site is located on the bank of the Kinema River, in the Archangelsk region (61°15′N 38°54′E). The dates obtained for this site range between 9,000–9,500 BP and 7,500–8,000 BP[167] and are expected to be revised to younger dates due to freshwater reservoir effects.

- Popovo2, male.

Supplementary Note 3

Y chromosomal haplogroup analysis

We were able to determine the Y chromosomal haplogroup by examining a set of diagnostic positions on chromosome Y using the ISOGG database (http://isogg.org/, accessed in 2016 March). In order to perform this analysis, we restricted our analysis to only include reads with a mapping quality higher than 30. Afterwards, we determined the haplogroups by identifying the most derived Y chromosomal SNP in our individual.
We caution that these haplogroup assignments are based partially on C-to-T and G-to-A polymorphisms at sites that may be affected by deamination.

Donkalnis4 (Kunda culture) could possibly be assigned as haplogroup **I** based on CTS10058: A->G (1x) and an upstream mutation for haplogroup CF: M3690: A->G (1x). But we caution that this assignment might be caused by false positive mutations since the coverage of this sample is very low that we don't have enough covered markers to verify the authenticity.

Spiginas1 (Narva culture) could be assigned as **I2a1a2a1a** based on L233:G→A (2x). This individual also has one upstream mutations for haplogroup I2a1a2a (L1286: G→A at 1x) and one mutation for I2a1 (PF4004: T→C at 1x) and I2a (L460: A→C at 1x).

Donkalnis7 (Narva culture) could be assigned as haplogroup **R1** based on P294:G->C (1x) and M306 (C->A) (1x). This individual also has two upstream mutations for haplogroup R: P224: C->T and M734:C->T.

Kretuonas2 (Narva culture) has a derived allele at I2a1b1:C→T, however only with coverage of 1x. Due to missing significance at that position we are not confident in this assignment. We were however able to find multiple upstream mutations assigning this individual to I2a1b (CTS176, CTS1293, CTS1802, CTS5375, CTS7218, and S2702). We are confident that the placement of this sample in Y chromosomal haplogroup **I2a1b** is correct.

Kretuonas5 (Narva culture) could be assigned as haplogroup **I** based on mutations PF3640 : T->A, PF3641 : T->C, FI2 : C->G,CTS6231 : C->T, FGC2412 : A->G, and Z16987:A->G. But we caution that this individual has an ancestral allele at PF3627.2, contradicting the haplogroup I assignment.

Kivisaare3 (Narva culture) could possibly be assigned as haplogroup K based on mutation P131: C->T, however only with coverage of 1x. Therefore we are not convinced that this represents the truth assigning this individual to K, due to missing significance at that position. This individual also has mutiple upstream mutations for haplogroup F: P145: G->A (1x), M235:T->G (1x) and M89:C->T (1x). Due to low coverage we refrain from assigning the haplogroup for this individual.

Tamula3 (Combed Ware culture) could be assigned as haplogroup **R1** based on mutations P286: C->T (1x) and P236: C->G (1x). This individual also has two upstream mutations for haplogroup R: M734: C->T (1x) and P285: C->A (1x).

Gyvakarai1 (Corded Ware complex) could be assigned as haplogroup **R1a1a1b** based on mutation S441: G->A (6x), upstream haplogroup R1a1a1 based on M417: G->A (2x) and Page7: C->T (3x), upstream haplogroup R1a1a based on M198: C->T (2x), L168: A->G (4x), M512: C->T (1x), and L449: C->T (2x), and upstream haplogroup R1a1 based on PF6234: C->T(1x) and M459: A->G (2x).

Kunila2 (Corded Ware complex) has a derived allele at R1a1a1b:C→T, however only with coverage of 1x. Due to missing significance at that position we are not confident in this assignment. We were able to find one upstream mutation assigning this individual to **R1a1a1** (Page7: C→T at 1x), two mutations assigning this individual to R1a1a (M198:C→T at 2x and M512:C→T at 1x) and one mutation to R1a1 (PF6234: C→T at 1x).

Spiginas2 (Corded Ware complex) could be assigned as **R1a1a1b** based on S441:G →A (3x). This individual also has multiple upstream mutations for haplogroup R1a1a (M515, L168, M512, M514, L449), R1a1 (PF6234 and L120) and R1a (L63 and L146).

Olsund (Late Neolithic without context) could be assigned as haplogroup **R1a1a1b** based on mutation S441:G->A (3x). This individual has three upstream mutatoions for haplogroup R1a1a: M515: T->A (1x), L168:A->G (1x), L449: C->T (1x), three upstream mutations for haplogroup R1a1: PF6234:C->T (1x), M459:A->G (2x) and M516: A->G (1x), and three mutations for haplogroup R1a: L145: C->A(6x), L62:A->G (8x), and L63:T->C (1x).

Turlojiske1 (Baltic Bronze Age) could be assigned as haplogroup **R1a1a1b** based on mutation S441:G->A (1x). This individual also has one upstream mutation R1a1: M459: A->G (1x) and one upstream mutation for haplogroup R1: P231: A->G (1x).

Turlojiske3 (Baltic Bronze Age) could be assigned as **R1a1a1b** based on S441:G→A (1x). This individual also has one upstream mutation for haplogroup R1a1a (L168:A →G at 1x), R1a1 (PF6234:C→T at 2x) and R1a (L62 and L63).

Turlojiske5 (Baltic Bronze Age) could only be assigned as haplogroup **CT** based on mutations M5612: C->T (1x), M5613: C->T (1x), M5632: G->A (1x), Z17706: G->T (1x), Z17713: T->C (1x), CTS5746: T->G (1x), M5751: A->G (2x), and M5823: C->T (1x).

Kivutkalns19 (Baltic Bronze Age) could be assigned as **R1a1a1b** based on S441:G →A (4x) and S224:C→T (1x). This individual also has two upstream mutations for haplogroup R1a1a1 (M417 and Page7) and two mutations for R1a1a (M515 and L449) and R1a1 (PF6234 and M459). We are confident that the placement of this sample in Y chromosomal haplogroup R1a1a1b is correct.

Kivutkalns25 (Baltic Bronze Age) could be assigned as **R1a1a1b** based on S441:G →A (3x). This individual also has one upstream mutation for haplogroup R1a1a1 (M417) and two mutations for R1a1a (M515 and L449) and R1a1 (M516 and M459).

We are confident that the placement of this sample in Y chromosomal haplogroup R1a1a1b is correct.

Kivutkalns153 (Baltic Bronze Age) could be assigned as haplogroup **R1b1a2** based on mutations L150.1:C->T (2x) and CTS11468: T->G (1x). This individual also has two upstream mutations for haplogroup R1: P238: G->A (1x) and P286: C->T (1x).

Kivutkalns164 (Baltic Bronze Age) could tentatively be assigned as haplogroup **R1a1** based on mutation PF6234:C->T, however only with coverage of 1x. This individual also has one upstream mutation for haplogroup R1: P238: G->A (2x) and one upstream mutation for haplogroup R: P285: C->A (1x).

Kivutkalns194 (Baltic Bronze Age) has a derived allele at **R1a1a**-L168:A→G. We were able to find one upstream mutation assigning this individual to R1a (L62:A→G at 2x) and one mutation assigning this individual to R1 (P286: C→T at 2x).

Kivutkalns209 (Baltic Bronze Age) has a derived allele at R1a1a1-Page7:C→T, however only with coverage of 1x. Therefore we are not convinced that this represents the truth assigning this individual to R1a1a1, due to missing significance at that position. We were however able to find two upstream mutation assigning this individual to R1a1a (M515: T→A at 1x, L168:A→G at 4x) and multiple mutations assigning this individual to R1a1 (PF6234, L120 and M459). We are confident that the placement of this sample in Y chromosomal haplogroup **R1a1a** is correct.

Kivutkalns222 (Baltic Bronze Age) has a derived allele at R1a1a1-M417:G→A, however only with coverage of 1x. Therefore we are not convinced that this represents the truth assigning this individual to R1a1a1, due to missing significance at that position. We were able to find one upstream mutation assigning this individual to R1a1a (M512: C→T at 1x), one mutation assigning this individual to R1a1 (M459:A →G at 1x) and one mutation to R1a (L62: A→G at 4x) and are confident with an assignment to **R1a1**.

Due to low coverage no assignment could be made for **Popovo2** (EHG).

Supplementary References

1. Lazaridis, I. *et al.* Ancient human genomes suggest three ancestral populations for present-day Europeans. *Nature* **513**, 409-413, doi:10.1038/nature13673 (2014).
2. Haak, W. *et al.* 2015. Massive migration from the steppe was a source for Indo-European languages in Europe. *Nature* 522, 207-211, doi:10.1038/nature14317.
3. Kriiska, A. 2009. The beginning of farming in the Eastern Baltic. P.M. Dolukhanov, G.R. Sarson & A.M. Shukurov (eds), *East European Plain on the Eve of Agriculture.* BAR IS 1964. Oxford: Archaeopress, 159–179.
4. Lang, V. 2007. *The Bronze and Early Iron Ages in Estonia*. Estonian
5. Loze I. *1993* The Early Neolithic *in* Latvia. The Narva Culture. *Acta Archaeologica* 63, 1992, 119-140.
6. Zagorska I. 1999. The earliest settlement of Latvia. In: Miller U, Hackens T, Lang V, Raukas A, Hicks S, editors. Environmental and Cultural History of the Eastern Baltic Region. PACT 57:131–56.
7. Antanaitis-Jacobs, I. & Girininkas, A. 2002 Periodization *and* chronology of the Neolithic in Lithuania. *Archaeologia Baltica* 5, 9–39.
8. Antanaitis-Jacobs, I., Richards, M., Daugnora, L., Jankauskas, R., Ogrinc, N. 2009. Diet in early Lithuanian prehistory and the new stable isotope evidence. *Archaeologia Baltica* 12, 12-30
9. Kriiska, A. & Tvauri, A. 2002. *Eesti muinasaeg*. Tallinn: Avita.
10. Kriiska, A. 2015. Foreign Materials and Artefacts in the 4th and 3rd Millennia BCE Estonian Comb Ware Complex. In: Espak, P., Läänemets, M., Sazonov, V. (Ed.). *When Gods Spoke. Researches and Reflections on Religious Phenomena and Artefacts. Studia in honorem Tarmo Kulmar.* (107−124). Tartu: Tartu University Press. (Studia Orientalia Tartuensia, Series Nova; VI).
11. Indreko, R. 1948. Die mittlere Steinzeit in Estland. Stockholm, 427
12. Jaanits, L., Laul, S., Lõugas, V. & Tõnisson, E. 1982. *Eesti esiajalugu*. Tallinn, Eesti Raamat.
13. Lõugas, L. 1996. Stone Age fishing strategies in Estonia – what did they depend on? *Archaeofauna*, 5, 101–109.
14. Kriiska, A., Hertell, E. & Manninen, M.A. 2011. Stone Age Flint Technology in South-Western Estonia: Results from the Pärnu Bay Area. In: T. Rankama (ed.) *Mesolithic Interfaces. Variability in Lithic Technologies in Eastern Fennoscandia.* Monographs of the Archaeological Society of Finland 1, 64–93.
15. Kriiska, A. 2009. The beginning of farming in the Eastern Baltic. P.M. Dolukhanov, G.R. Sarson & A.M. Shukurov (eds), *East European Plain on the Eve of Agriculture.* BAR IS 1964. Oxford: Archaeopress, 159–179.
16. Zagorska, I. 2006. Radiocarbon chronology of the Zvejnieki burials. In: L. Larsson & I. Zagorska (eds.). *Back to the Origin. New reserch in the*

Mesolithic-Neolithic Zvejnieki cemetery and environment, northern Latvia. Acta Archaeologica Lundensia. Series in 8°, No. 52. Stockholm: Almqvist & Wiskell International, 91–113.

17. Mannermaa, K., Zagorksa, I., Junger, H. & Zarina, G. 2007. New radiocarbon dates of human and bird bones from Zvejnieki Stone Age burial ground in northern Latvia. *Before Farming*, 1–11.
18. Jaanits, L.1965. Über die Ergebnisse der Steinzeitforschung in Sowjetestland. *Finskt Museum*, LXXII, 5–46.
19. Loze I. *1993* The Early Neolithic *in* Latvia. The Narva Culture. *Acta Archaeologica* 63, 1992, 119-140.
20. Rimantienė, R., *1994* Die Steinzeit in Litauen*, Bericht der Römisch-Germanischen Kommission* 75, 27-146.
21. Kriiska, A. & Lõugas, L. 1999. Late Mesolithic and Early Neolithic seasonal settlment at Kõpu, Hiiumaa Island, Estonia. In: U. Miller, T. Hackens, V. Lang, A. Raukas, S. Hicks (eds.). *Environmental and Cultural History of the Eastern Baltic Region.* Rixensart: UNESCO, 157–172.
22. Nordqvist, K. and Kriiska, A. (2015). Towards Neolithisation. The Mesolithic-Neolithic transition in the central area of the eastern part of the Baltic Sea. In: Jacek Kabaciński, Sönke Hartz, Daan C. M. Raemaekers and Thomas Terberger (Ed.). The Dąbki Site in Pomerania and the Neolithisation of the North European Lowlands (c. 5000–3000 calBCE). (537−556). Leidorf: Verlag Marie Leidorf GmbH . Rahden/Westf. (Archaologie und Geschichte im Ostseeraum; 8).
23. Lõugas, L., Kriiska, A. & Maldre, L. 2007. New dates for the Late Neolithic Corded Ware Culture burials and early husbandry in the East Baltic region. *Archaeofauna,* 16, 21–31.
24. Allentoft, M. E. *et al.* 2015. Population genomics of Bronze Age Eurasia. *Nature* 522, 167-172, doi:10.1038/nature14507.
25. Tõrv, M. 2016. Persistent Practices. A Multidiscilplinary Study of Hunter-Gatherer Mortuary Remains from c. 6500-2500 BC, Estonia. DISSERTATIONES ARCHAEOLOGIAE UNIVERSITATIS TARTUENSIS, 5. Tartu, Tartu University Press.
26. Lang, V. & Kriiska, A. 2007. The Final Neolithic and Early Bronze Age Contacts between Estonia and Scandinavia. In: Fransson, U., Svedin, M., Bergerbrant, S. & Androshchuk, F. (eds*.). Cultural interaction between east and west. Archaeology, artefacts and human contacts in northern Europe*. Stockholm: Stockholm University, 107–112.
27. Jaanits, L. 1979. Die Neolithische Siedlung Kõnnu auf der Insel Saaremaa. *Eesti NSV Teaduste Akadeemia Toimetised,* 28, (4). Tallinn, 363–367.
28. Kriiska, A. 2007. Saaremaa kiviaeg. K. Jänes-Kapp, E. Randma & M. Soosaar (eds), *Saaremaa. Ajalugu. Majandus. Kultuur. 2*. Tallinn: Koolibri, 9–36.
29. Poska, A. & Saarse, L. 2002. Vegetation development and introduction of agriculture to Saaremaa Island, Estonia: the human response to shore displacement. *The Holocene*, 12(5), 555–568.

30. Saarse, L., Vassiljev, J. & Rostentau, A. 2009. Litorina Sea shore displacement of the island of Saaremaa, Estonia. *Polish Geological Institute Special Papers*, 25, 59–66.
31. Saarse, L., Vassiljev, J. & Rosentau, A. 2009. Ancylus Lake and Litorina Sea transition on the Island of Saaremaa, Estonia: pilot study. *Baltica*, 22(1), 51–62.
32. Kriiska, A. 1997. Aruanne arheoloogilisest inspektsioonist Ihastes (Tartu-Maarja khk.) 22.–27. sept. 1997. Tartu. (Manuscript in TÜ AK)
33. Allmäe, R. 2011. Stone Age quadruple burial in Veibri village, Tartumaa Estonia – some anthropological data. *Arheoloģija un Etnogrāfija,* 25, 182–189.
34. Kriiska, A., Lõugas, L., Lõhmus, M., Mannermaa, K. & Johanson, K. 2007. New AMS dates from Estonian Stone Age burials sites. *Estonian Journal of Archaeology*, 11(2), 83–121.
35. Ottow, B. 1911. Das neolithische Grabfeld von Kiwisaar an der Phale (Nordlivland). *Sitzungsberichte der Gelehrten Estnischen Gesellschaft, 1910*. Jurjew-Dorpat, 148– 160.
36. Bolz, M. 1914. Das neolithische Gräberfeld von Kiwisaare in Livland. *Baltische Studien zur Archäologie und Geschichte. Arbeiten des Baltischen Vorbereitenden Komitees für den XVI. Archäologischen Kongress in Pleskau 1914*. Berlin: Gesellschaft für Geschichte und Altertumskunde der Ostseeprovinzen Russlands, 15–32.
37. Tallgren, A. M. 1922. Zur Archäologie Eestis I. Vom Anfang der Besiedlung bis etwa 500 n. Chr. *Acta et Commentationes Universitatis Tartuensis (Dorpatensis)*, B III(6). Dorpat.
38. Kriiska, A., Allmäe, R., Lõhmus, M. & Johanson, K. 2004. Archaeological investigation at the settlement and burial site of Kivisaare. *Archaeological Fieldwork in Estonia 2003*, 29–44.
39. Kriiska, A. & Lõhmus, M. 2005. Archaeological fieldwork on Kivisaare Stone Age burial ground and settlement site. *Archaeological Fieldwork in Estonia 2004, 31-43.*
40. Tõrv, M. & Meadows, J. 2015. Radiocarbon dates and stable isotope data from the Early Bronze Age burials in Riigiküla I and Kivisaare settlement sites, Estonia. *Radiocarbon*, 57(4), 645–656.
41. Jaanits, L. 1965. Aruanne arheoloogilistest kaevamisetst Kivisaare kalmistul Viljandi rajoonis end. Kolga-Jaani kihelkonnas 29.VI 1962. a. ja 6.–19. VII 1965. (Manuscript in TLU AI).
42. Mark, K. Personal Archive. *Antropoloogiliste kogude kataloog.* Archaeological Archive at Tallinn University (TLU AI, F18).
43. Lõugas, L., Lidén K, & Nelson, D.E. 1996. Resource utilisation along the Estonian coast during the Stone Age. T. Hackens, S. Hicks, V. Lang, U. Miller & L. Saarse (eds), *Coastal Estonia: Recent Advances in Environmental and Cultural History*. PACT 51. Strasbourg: Council of Europe, Rixensart: PACT Belgium, 399–420.

44. Hausmann, R. 1904. Ueber Gräber aus der Steinzeit im Ostbaltikum: Grabfunde in Woisek und Kölljal. *Sitzungs-Berichte der Gelehrten Estnischen Gesellschaft*, 1903, 71–81.
45. Mannermaa, K. 2008. *The Archaeology of Wings. Birds and People in the Baltic Sea Region during the Stone Age*. Helsinki: Helda.
46. Ots, M. 2006. Merevaiguleiud Baltimaade kivi- ja pronksiaja muististes. MA dissertation. (anuscript in the Library of the University of Tartu.)
47. Allmäe, R. 2006. Tamula asulakoha matuste antropoloogilised määrangud. 17.10.2006. (Manuscript in TLU AI).
48. Lõhmus, M. 2005. Kammkeraamika kultuuride matused Eestis ning nende tõlgendusprobleemid. BA Thesis. Tartu-Narva. (Manuscript in Archaeology Department, University of Tartu)
49. Indreko, R. 1931. Aruanne kaevamistest Harjumaal Kose kihelkonnas 7. mail 1931. (Manuscript in the Institute of History of Tallinn University.)
50. Saadre, E. 1936. Aruanne Kose khk., Triigi vld., Ardu kl., Hansu-Mardi tl. maal leitud kiviaja luustiku kohta suvel 19. augustil 1936. (Manuscript in the Institute of History of Tallinn University.)
51. Moora, H. 1926. Aruanne kaevamistest Lüganuse khk. Sope kl. Metsvälja tl. maal 23. aug. 1926. a. (Manuscript in the Institute of History of Tallinn University.)
52. Indreko, R. 1933. Aruanne kaevamistest Lüganuse khk. Püssi vld. Sope kl. Metsvälja tl. maal 9.-13. VIII. 1933. (Manuscript in the Institute of History of Tallinn University.)
53. Rasmussen et al. 2015. Early Divergent Strains of Yersinia pestis in Eurasia 5,000 Years Ago. *Cell*, Volume 163, Issue 3, 571–582.
54. Jaanits, L. 1948. Aruanne kaevamistest Kursi khk-s ja vallas Kunila külas Mäe-Jaaniantsu e. Keldri talu piirides asuval Jaaniantsu mäel 5.-10. juunini 1948. (Manuscript in the Institute of History of Tallinn University.)
55. Jaanits, L. 1955. Neoliitilised asulad Eesti NSV territooriumil. H. Moora & L. Jaanits (eds), *Muistsed asulad ja linnused. Arheoloogiline kogumik*. Tallinn: Eesti Riiklik Kirjastus, 176–201.
56. Jaanits, L. 1976. Ausgrabungen der neolithischen Siedlung in Kääpa. ENSV TA Toimetised. Uhiskonnateadused, 25, 1, 45-48
57. Jaanits, L. 1954. Aruanne arheoloogilistest proovikaevamistest muistsel asulakohal Narva linnas Joaorus 8.–25. juunini 1954. a. (Manuscript in TLU AI, 1-95-37)
58. Jaanits, L. & Liiva, A. 1973. Eesti vanima ajaloo kronoloogia ja radiosüsinukumeetod. N. Alumäe (ed), *Eesti NSV Teaduste Akadeemia aastail 1965–1972*. Tallinn: Akadeemia, 157–161.
59. Rosentau, A. Muru, M., Kriiska, A., Subetto, D.A., Vassiljev, J., Hang, T., Gerasimov, D., Nordqvist, K., Ludikova, A., Lõugas, L., Raig, H., Kihno, K., Aunap, R. & Letyka, N. 2013. Stone Age settlement and Holocene shore displacement in the Narva-Luga Klint Bay area, eastern Gulf of Finland. Boreas. *An International Journal of Quaternary Research*, 912–931.

60. Duderis, K., 2015. Tyrimai prie Širvėnos ežero ir Apaščios upės santakos. In G. Zabiela (ed.). *Archeologiniai tyrinėjimai Lietuvoje 2014 metais*. Vilnius: BALTOprint. *In press*

61. Butrimas, A. 2012. *Donkalnio ir Spigino Mezolito – Neolito kapinynai. Seniausi laidojimo paminklai Lietuvoje*. Vilnius: Vilniaus dailės akademijos leidykla.

62. Butrimas, A., Kazakevičius, V., Česnys, G., Balčiūnienė, I., Jankauskas, R. 1985. Ankstyvieji Virvelinės keramikos kultūros kapai Lietuvoje. In R. Volkaitė – Kulikauskienė, R. Rimantienė & G. Česnys (eds.). Akmens amžiaus gyvenvietės ir kapinynai: 14 – 24. *Lietuvos Archeologija 4.* Vilnius: Mokslas.

63. Kunskas, R., Butrimas, A., Česnys, G., Balčiūnienė, I., Jankauskas, R. 1985. Duonkalnis: vėlyvojo neolito gyvenvietė, alkas ir kapinynas. In R. Volkaitė – Kulikauskienė, R. Rimantienė & G. Česnys (eds.). Akmens amžiaus gyvenvietės ir kapinynai: 25 – 66. *Lietuvos Archeologija 4*. Vilnius: Mokslas.

64. Tebelškis, P., Jankauskas, R.. 2006. The Late Neolithic Grave at Gyvakarai in Lithuania in the Context of Current Archaeological and Anthropological Knowledge. In V. Žulkus, V. Kazakevičius & A. Girininkas (eds.). *Archaeologia Baltica 6*: 8 – 20. Vilnius: Petro ofsetas

65. Girininkas, A., Česnys, G., Balčiūnienė, I., Jankauskas, R. 1985. Kretuono I-os gyvenvietės vidurinio neolito kapai. In R. Volkaitė – Kulikauskienė, R. Rimantienė & G. Česnys (eds.): Akmens amžiaus gyvenvietės ir kapinynai: 5 – 14. *Lietuvos Archeologija 4.* Vilnius: Mokslas.

66. Kazakevičius, V. 1993. *Plinkaigalio kapinynas. Monografija*. Lietuvos archeologija 10. Vilnius, Vilniaus mokslo ir enciklopedijų leidykla.

67. Ramsey, C. B., Pettitt, P. B., Hedges, R. E. M., Hodgins, H. W. L., Owen, D. C. 2000. Radiocarbon dates from the Oxford AMS System. Archaeometry datelist 29. *Archaeometry* 42 (1), 243 – 254. Blackwell Publishing Ltd.

68. Merkevičius, A. 2012. Turlojiškė Archaeological Complex. In G. Zabiela, Z. Baubonis, E. Marcinkevičiūtė (eds.). *Archeological Investigations in Independent Lithuania 1990-2010*, 12 – 16. Vilnius.

69. *Lietuvs Aidas*, 1930.09.13, no.208.

70. Žilinskas, J. 1931. *Akmens period (mesolithicum-neolithicum) žmogus Žemaitijoj ir Suvalkijoj, jo kilmė ir jojo ainiai*, 9 – 29, Kaunas

71. Česnys, J. 2001. Reinventing the Bronze Age man in Lithuania: Skulls from Turlojiškė. In *Acta medicina Lithuanica*, 4 – 6, Vilnius.

72. Vasks A.(2010); Latvia as part of a sphere of contacts in the Bronze Age. *Archaeologia Baltica* 13: pp. 153-160.

73. Graudonis J. 1989. Hillforts in lower reaches of Daugava. Riga, Zinātne.

74. Oinonen, M., Vasks, A., Zarina, G., Lavento, M. 2013. Stones, Bones, and Hillfort: Radiocarbon Dating of Kivutkalns Bronze-Working Center. RADIOCARBON55, 3 pp. 1252-1264

75. Denisova R., Gravere R., Graudonis J.(1985) Bronze age cemetery of Ķivutkalns. Riga. Zinatne.

76. Oinonen M et al. 2017. Manuscript on ^{14}C datings, stable isotopic analyses and reservoir effects on the samples of Kivutkalns site, in preparation.
77. Larsson, L. 1994. The Earliest Settlement in Southern Sweden. Late Paleolithic Settlement Remains at Finjasjön, in the North of Scania. *Current Swedish Archaeology* 2: 159–178.
78. Larsson, L., R. Liljegren, O. Magnell and J. Ekström. 2002. Archeo-faunal aspects of bog finds from Hässleberga, southern Scania, Sweden. In: V. B. Eriksson and B. Bratlund (eds.). *Recent Studies in the Final Palaeolithic of the European Plain.* Jutland Archaeological Society Publications Vol. 39, Jutland Archaeological Society: 61-74.
79. Knarrström, B., 2004. The introduction of culture. 12000–7500 BC. *In*: M. Andersson, P. Karsten, B. Knarrström and M. Svensson, eds. *Stone age Scania: significant places dug and read by contract archaeology.* Riksantikvarieämbetet, Stockholm: 21-70.
80. Fischer, A, Clemmensen, L. B., Donahue, R., Heinemeier, J., Lykke-Andersen, H., Lysdahl, P., Mortensen, M. F., Olsen, J. & Petersen, P. V. 2013, Late Palaeolithic Nørre Lyngby – a northern outpost close to the west coast of Europe. *Quartaer* 60: 137-162.
81. Nordqvist, B. 2000. C*oastal adaptions in the Mesolithic. A study e of coastal sites with organic remains from the Boreal and Atlantic periods in Western Sweden.* Göteborg University, Göteborg.
82. Bjerck, H.B. 2009. Colonizing Seascapes: Comparative Perspectives on the Development of Maritime Relations in Scandinavia and Patagonia. *Arctic Anthropology* 46 (1-2): 118-131.
83. Schmitt, L., S. Larsson, J. Burdukiewicz, J. Ziker, K. Svedhage, J. Zamon & H. Steffen. 2009. Chronological insights, cultural change, and resource exploitation on the west coast of Sweden during the Late Palaeolithic/Early Mesolithic transition. *Oxford Journal of Archaeology* 28(1): 1-27.
84. Fuglestvedt, I. 2012. The Pioneer Condition on the Scandinavian Peninsula: the Last Frontier of a 'Palaeolithic Way' in Europe. *Norwegian Archaeological Review* 45 (1): 1-29.
85. Breivik, H. 2014. Palaeo-oceanographic development and human adaptive strategies in the Pleistocene–Holocene transition: A study from the Norwegian coast. *The Holocene* 2014. DOI: 10.1177/0959683614544061
86. Kankaanpää, J. & T. Rankama. 2011. Spatial Patterns of the Early Mesolithic Sujala Site, Jaksland, L. & P. Persson. 2014. *E18 Brunlanesprosjektet, Bind I, Forutsetninger og kulturhistorisk sammenstillning*. Varia 79, Museum of Cultural History, University of Oslo.
87. Möller, P., O. Östlund, L. Barnekow, P. Sandgren, F. Palmbo & E. Willerslev. 2012. Living at the margin of the retreating Fennoscandian Ice Sheet: The early Mesolithic sites at Aareavaara, northernmost Sweden. *The Holocene* 23 (1): 104-116.
88. Hallgren, F. & E. Fornander. in press. Skulls on stakes and skulls in water. Mesolithic mortuary rituals at Kanaljorden, Motala, Sweden 7000 BP, in:

Grünberg, J. (ed.) *Mesolithic burials - Rites, symbols and social organisation of early postglacial communities.* Tagungen des Landesmuseums für Vorgeschichte Halle, Halle: 161-174.
89. Knutsson, H. & Knutsson, K. 2012. The postglacial colonization of humans, fauna and plants in northern Sweden. *Arkeologi i Norr* 13, 1-28.
90. Sørensen, M., Rankama, T., Kankaanpää, J., Knutsson, K., Knutsson, H., Melvold, S., Eriksen, B. V. & Glørstad, H. 2013. The first eastern migrations of people and knowledge into Scandinavia: Evidence from studies of Mesolithic Technology. *Norwegian Archaeological Review* 46 (1): 1-38.
91. Hallgren, F. 2004. The introduction of ceramic technology around the Baltic Sea in the 6th millennium. In: Knutsson, H. (ed.). *Coast to coast - landing.* Uppsala University, Uppsala: 123-142.
92. David, É. 2009. Show me how you make your hunting equipment and I will tell you where you come from: Technical traditions, an efficient means to characterize cultural identities. In: S. McCartan, R. Schulting, G. Warren & P. Woodman (eds.), *Mesolithic Horizons; Papers presented at the Seventh international conference on the Mesolithic in Europe.* Oxbow Books, Exeter: 362-367
93. Bergsvik, K-A. & É David. 2015. Crafting bone tools in Mesolithic Norway, a regional eastern-related know-how. *Journal of European Archaeology* 18 (2): 190-221.
94. Price, T. D. 2000. The introduction of farming in Northern Europe. In: Price, T. D. (ed.). *Europe's First Farmers.* Cambridge University Press, Cambridge: 260-300.
95. Fischer, A. 2002. Food for feasting? An evaluation of explanations of the neolithisation of Denmark and southern Sweden. In: Fischer, A. & Kristiansen, K. (eds.). *The Neolithisation of Denmark. 150 Year Debate.* J. R. Collis Publications, Sheffield: 343-393.
96. Hallgren, F. 2008. *Identitet i praktik. Lokala, regionala och överregionala sociala sammanhang inom nordlig trattbägarkultur.* Uppsala Universitet, Uppsala.
97. Hallgren, F. 2011. The early 'Trichterbecher' of Mälardalen, eastern Central Sweden. In: Hartz, S., Lüth, F. & Terberger, T. (ed.). *Early Pottery in the Baltic – Dating, Origin and Social Context.* Bericht der Römisch-Germanische Kommission 89. Frankfurt: 111-134.
98. Sørensen, L. 2014. From hunter to farmer in Northern Europe. Migration and adaptation during the Neolithic and Bronze Age. Acta Archaeologica 85:1. Wiley, Oxford.
99. Andersson, M., Artursson, M. & Brink, K. 2016. Early Neolithic Landscape and Society in Southwest Scania – New Results and Perspectives. *Journal of Neolithic Archaeology* 18: 23-114.
100. Koch, E. 1998. *Neolithic bog pots from Zealand, Møn, Lolland and Falster.* Det Kongelige Nordiske Oldskriftselskab, København.
101. Jennbert, K. 2011. Ertebølle pottery in southern Sweden – a question of

handcraft, networks and creolisation in a period of neolithisation. In: Hartz, S., Lüth, F. & Terberger, T. (ed.). Early Pottery in the Baltic – Dating, Origin and Social Context. Bericht der Römisch-Germanische Kommission 89. Frankfurt: 89-110.

102. Sjögren, K-G. 2011. C-14 chronology of scandinavian megalithic tombs. In: García Sanjuán, L.; Scarre, C. & Wheatley, D. W. (eds.). *Exploring Time and Matter in Prehistoric Monuments: Absolute Chronology and Rare Rocks in European Megaliths.* Menga, Journal of Andalusian Prehistory, Monograph 1: 103-119.

103. Craig, O. E., Steele, V. J., Fischer, A. Hartz, S., Andersen, S. H., Donohoe, P., Glykou, A., Saul, H., Jones, D. M., Koch, E. and Heron, C. P. 2011. Ancient lipids reveal continuity in culinary practices across the transition to agriculture in Northern Europe. *PNAS* 108(44): 17910-17915.

104. Isaksson, S. & Hallgren, F. 2012. Lipid residue analyses of Early Neolithic funnel-beaker pottery from Skogsmossen, eastern Central Sweden, and the earliest evidence of dairying in Sweden. *Journal of Archaeological Science* 39: 3600-3609.

105. Gron K. J., Montgomery J., Rowley-Conwy P. 2015. Cattle Management for Dairying in Scandinavia's Earliest Neolithic. *PLoS ONE* 10(7): e0131267. doi:10.1371/journal.pone.0131267

106. Hjelle, K. L., Hufthammer, A. K. & Bergsvik, K. A. 2006. Hesitant hunters: a review of the introduction of agriculture in western Norway. *Environmental Archaeology* 11(2): 147-170.

107. Østmo, E. 2007. The Northern periphery of the TRB. Graves and Ritual Deposits in Norway. *Acta Archaeologica* 78:2: 111-142.

108. Bergsvik, K. A. & Østmo, E. 2011. The experienced Axe. Chronology, condition and context of TRB-axes in Western Norway. In: Davis, V. & M. Edmonds (eds.). *Stone Axe Studies III.* Oxbow Books, Oxford: 1-20.

109. Glørstad, H. 2012. The Northern Province? The Neolithisation of Southern Norway. In: Glørstad, H. & C. Prescott (eds.) *Neolithisation as if history mattered. Processes of Neolithisation in North-Western Europe*. Bricoleur Press, Lindome: 135-168.

110. Solheim, S. 2012. *Lokal praksis og fremmed opphav. Arbeidsdeling, sosiale relasjoner og differensiering i østnorsk tidligneolitikum.* Oslo Universitet, Oslo.

111. Reitan, G. & Persson, P. 2014. *Seinmesolittiske, neolittiske og yngre lokaliteter i Vestfold og Telemark.* Vestfoldbaneprosjektet: Arkeologiske undersøkelser i forbindelse med ny jernbane mellom Larvik og Porsgrunn. Bind 2. Kulturhistorisk museum, Oslo.

112. Andersen, S. H. 2011. Kitchen middens and the early pottery in Denmark. In: Hartz, S., Lüth, F. & Terberger, T. (ed.). *Early Pottery in the Baltic – Dating, Origin and Social Context.* Bericht der Römisch-Germanische Kommission 89. Frankfurt: 193-215.

113. Hartz, S. 2011. From pointed bottom to round and flat bottom – tracking early

pottery from Schleswig-Holstein. eIn: Hartz, S., Lüth, F. & Terberger, T. (ed.). Early Pottery in the Baltic – Dating, Origin and Social Context. Bericht der Römisch-Germanische Kommission 89. Frankfurt: 241-276.
114. Stafford, M. 1999. *From forager to farmer in flint. A lithic analysis of the prehistoric transition to agriculture in Southern Scandinavia.* Aarhus University Press, Aarhus.
115. Hallgren, F. 2003. My place or yours?. In: Larsson, L., Kindgren, H., Knutsson, K., Loeffler, D. & Åkerlund, A (eds.). *Mesolithic on the move.* Oxbow books, Oxford: 592-599.
116. Fischer, A. & Kristiansen, K. (eds.). 2002. *The Neolithisation of Denmark. 150 Year Debate.* J. R. Collis Publications, Sheffield.
117. Rowley-Conwy, P. 2004 'How the West was lost: a reconsideration of agricultural origins in Britain, Ireland and southern Scandinavia.', *Current Anthropology* 45(S4): 83-113.
118. Rowley-Conwy, P. A. 2011. Westward Ho! The spread of agriculture from Central Europe to the Atlantic. *Current Anthropology* 52(S4): 431-451.
119. Malmer, M. P. 2002. *The Neolithic of South Sweden. TRB, GRK, and STR.* Almqvist & Wiksell International, Stockholm.
120. Sjögren, K-G. 2010. Megaliths, Landscapes and Identities: the case of Falbygden, Sweden. In: M. Furholt, F. Lüth & J. Müller (eds.): *Megaliths and Identities.* Rudolf Habelt, Bonn: 155-166.
121. Lidén, K. 1995. *Prehistoric diet transitions: an archaeological perspective.* Stockholm University, Stockholm.
122. Sjögren, K-G. 2003. *"Mångfalldige uhrminnes grafvar…" Megalitgravar och samhälle i Västsverige.* Göteborgs universitet, Göteborg.
123. Larsson, Å. M. 2009. *Making and Breaking Bodies and Pots. Material and Ritual Practices in South Sweden in the Third Millennium BC.* Uppsala universitet, Uppsala.
124. Hallgren, F. 2009. Early pottery among hunter-horticulturalists and hunter-gatherers in central Fenno-Scandinavia. In: Gheorghiu, D. (ed.). *Early Farmers, Late Foragers, and Ceramic Traditions: On the Beginning of Pottery in the Near East and Europe.* Cambridge Scholars Publishing, Cambridge: 215-238.
125. Edenmo, J. & Heimdahl, J. 2012. Gropkeramiskt jordbruk på Södertörn? In: Kihlstedt, B (ed.). Sittesta – en gropkeramisk boplats under 800 år. Riksanikvarieämbetet, Stockholm.
126. Andersson, H. 2013. *Stenålder i Åby, bland gravar och gropkeramik.* Arkeologikonsult, Upplands Väsby.
127. Segerberg, A. 1999. *Bälinge mossar. Kustbor i Uppland under yngre stenålder.* Uppsala universitet, Uppsala.
128. Storå, J. 2001. *Reading bones. Stone Age hunters and seals in the Baltic.* Stockholm University, Stockholm.
129. Eriksson, G. 2004. Part-time farmers or hard-core sealers? Västerbjers studied by means of stable isotope analysis. *Journal of Anthropological Archaeology*

23(2): 135-162.
130. Fornander, E., Eriksson, G. & Lidén, K. 2008. Wild at heart: Approaching Pitted Ware identity, economy and cosmology through stable isotopes in skeletal material from the Neolithic site Korsnäs in Eastern Central Sweden. *Journal of Anthropological Archaeology* 27(3): 281-297.
131. Olson, C. 2008. *Neolithic fisheries: Osteoarchaeology of fish remains in the Baltic Sea Region*. Stockholm University, Stockholm.
132. Larsson, Å. M. 2009. Pots, Pits, and People. Hunter-Gatherer Pottery Traditions in Neolithic Sweden. In: Gheorghiu, D. (ed.) *Early Farmers, Late Foragers, and Ceramic Traditions: On the Beginning of Pottery in the Near East and Europe.* Cambridge Scholars Publishing, Cambridge: 239-270.
133. Hallgren, F. 2012. Om gropkeramik och dess relation till äldre keramikhantverkstraditioner kring Östersjön. In: P. Bratt & R. Grönwall (eds.). *Gropkeramikerna. Rapport från ett seminarium 2011.* Stockholms läns museum, Stockholm: 31-43.
134. Meinander, C. F. 1957. Kolsvidja. *Finska Fornminnesföreningens tidskrift* 58 (1957): 185–213.
135. Brøgger, A. W. 1906. *Studier over Norges stenalder. 1, Øxer uden skafthul fra yngre stenalder fundne i det sydøstlige Norge.* Dybwad, Christiania.
136. Taffinder, J. 1998. *The allure of the exotic. The social use of non-local raw materials during the Stone Age in Sweden.* Uppsala University, Uppsala.
137. Browall, H. 1991. Om förhållandet mellan trattbägarkultur och gropkeramisk kultur. In: Browall H., P. Persson & K.-G. Sjögren (eds.). *Västsvenska stenåldersstudier.* Göteborgs universitet, Göteborg: 111-142.
138. Stenbäck, N. 2003. *Människorna vid havet. Platser och keramik på Ålandsöarna perioden 3500-2000 f.Kr.* Stockholms universitet, Stockholm.
139. Timofeev, V. I. 2000. On the problem of the Scandinavian Pitted Ware Origin and the definition of the eastern component in this process. In: Jaanits, L. & Lang, V. (eds.). De temporibus antiquissimis ad honorem Lembit Jaanits. Muinasaja teadus 8. Teaduste Akadeemia Kirjastus, Tallinn: 209-222.
140. Brink, K. 2009. *I palissadernas tid: om stolphål och skärvor och sociala relationer under yngre mellanneolitikum.* Lunds universitet, Lund.
141. Kristiansen, K. 1991. Prehistoric Migrations – the case of the Single Grave and Corded Ware Cultures. *Journal of Danish Archaeology* 8: 137–151.
142. Damm, C. 1993. The Danish Single Grave Culture - Ethnic Migration or Social Construction? *Journal of Danish Archaeology* 10: 199–204.
143. Hallgren, F. 2000. Lämningar från stridsyxekulturen på Fågelbacken, Hubbo sn, Västmanland. *Tor* 30: 5-33.
144. Baudou, E. 1992. *Norrlands forntid – ett historiskt perspektiv*. Wiken, Höganäs.
145. Knutsson, K. 1988. *Making and using stone tools. The analysis of the lithic assemblages from Middle Neolithic sites with flint in Västerbotten, northern Sweden*. Uppsala University, Uppsala.
146. Olausson, D, Hughes, R. & Högberg, A. 2012. A New Look at Bjurselet. The

Neolithic Flint Axe Caches from Västerbotten, Sweden using non-destructive energy dispersive x-ray fluorescence analysis for provenance determination. *Acta Archaeologica* 83: 83-103.

147. Schierbeck, A. 1994. *Hedningahällan, en undersökning för att skydda och vårda*. Riksantikvarieämbetet, Stockholm.
148. Holm, L. 2006. *Stenålderskust i norr. Bosättning, försörjning och kontakter i södra Norrland.* Umeå University, Umeå.
149. Gustafsson, P. & Spång, L. G. 2007. *Stenålderns stationer. Arkeologi i Botniabanans spår.* Riksantikvarieämbetet, Stockholm.
150. Larsson, Å. M. & Graner, G. 2010. More than meets the eye. Pottery craft in transition at the end of the Middle Neolithic in Eastern Sweden. In: Å. M. Larsson & L. Papmehl-Dufay (eds.). *Uniting Sea II. Stone Age Societies in the Baltic Sea Region.* OPIA 51. Department of Archaeology and Ancient History, Uppsala University: 213-247.
151. Hallgren, F. 1996. En mellanneolitisk skalle från Hälsingland. *Fjölnir* 15(1): 5-10.
152. Hallgren, F. 2011. Mesolithic skull depositions at Kanaljorden, Motala, Sweden. Current Swedish Archaeology 19: 244-246.
153. Gummesson, S. & A. Kjellström. *Manuscript.* Osteologisk rapport av humana skelettfynd från Kanaljorden, Motala, Östergötland. OFL, Stockholm.
154. Eriksson, G., Frei, K. M., Howcroft, R., Gummesson, S., Molin, F., Lidén, K., Frei, R. & Hallgren, F. 2016. Diet and mobility among Mesolithic hunter-gatherers in Motala (Sweden) – the isotope perspective. *Journal of Archaeological Science: Reports.* http://dx.doi.org/10.1016/j.jasrep.2016.05.052.
155. Nilsson, M-L & Nilsson, L. 2003. Ett källsprång i Saxtorp. In: Svensson, M. (ed.): *I det neolitiska rummet.* Riksantikvarieämbetet, Lund: 242-295.
156. Regnell, M. & K-G. Sjögren. 2006. Vegetational development. In: Sjögren K-G. (ed.). *Ecology and Economy in Stone Age and Bronze Age Scania.* Riksantikvarieämbetet, Lund: 40-79.
157. Andersson, M. 2004. *Making place in the landscape: early and middle Neolithic societies in two west Scanian valleys.* Riksantikvarieämbetet, Lund.
158. Regnell, M. & K-G. Sjögren. 2006a. Introduction and development of agriculture. In: Sjögren K-G. (ed.). *Ecology and Economy in Stone Age and Bronze Age Scania.* Riksantikvarieämbetet, Lund: 106-169.
159. Gummesson, S. *manuscript.* Osteologisk köns- och åldersbedömning av kraniefynd från Ölsund, Hälsningland. OFL, Stockholm.
160. Miettinen A., Sarmaja-Korjonen K., Sonninen E., Jungner H., Lempiäinen T., Ylikoski K., Mäkiaho J.-P. and Carpelan C. 2008. The Palaeoenvironment of the ‘Antrea Net Find’. In: Lavento M, ed., *Karelian Isthmus. Stone Age studies in 1998–2003*. Iskos, Helsinki, 16: 71–87.
161. Tambets, K., Rootsi, S., Kivisild, T., Help, H., Serk, P., Loogväli, E. L., et al. 2004. The Western and Eastern Roots of the Saami - The Story of Genetic

"Outliers" Told by Mitochondrial DNA and Y Chromosomes. *American Journal of Human Genetics*, *74*(4), 661-682. DOI: 10.1086/383203
162. Jacobs K. 1995. Returning to Oleni' ostrov: Social, Economic, and Skeletal Dimensions of a Boreal Forest Mesolithic Cemetery. J Anthropol Archaeol 14: 359–403.
163. Gurina N.N. 1956. Oleneostrovski Mogil'nik. In *Materialy i Issledovaniya po Arkheologgi SSSR*. Moscow: Nauka, Akademia Nauk SSSR.
164. O'Shea J., Zvelebil M. 1984. Oleneostrovskii Mogilnik: Reconstructing Social and Economic Organisation of Prehistoric Hunter-Fishers in Northern Russia. J Anthropol Archaeol 1: 1–40.
165. Price, T.D. and Jacobs K. 1990. Olenii Ostrov: First radiocarbon dates from a major Mesolithic cemetery in Karelia, USSR. Antiquity, 64, pp 849-853.
166. Wood R. 2006. Chronometric and paleodietary studies at the Mesolithic and Neolithic burial ground of Minino, NW Russia. Dissertation for the MSc in archaeological Science. Oxford University.
167. Oshibkina S.V. 1999. Tanged Point Industries in the North-West of Russia. In: Kozlowski SK, Gurba J, Zaliznyak LL, editors. Tanged Points Cultures in Europe. Lublin.
168. R: A language and environment for Statistical Computing, R Core Team, R Foundation for Statistical Computing, Vienna, Austria (http://www.R-project.org) (2016).
169. Hadley, W. ggplot2: Elegant graphics for data analysis (https://cran.r-project.org/web/packages/ggmap/citation.html) (2009).
170. Kahle, D. & Wickham, H. ggmap: Spatial Visualization with ggplot2. *R Journal* 5 (https://cran.r-project.org/web/packages/ggplot2/citation.html) (2013).
171. Piličiauskas, G., Jankauskas, R., Piličiauskienė, G., Craig, O. E., Charlton, S., Dupras, T. 2017. The transition from foraging to farming (7000–500 cal BC) in the SE Baltic: A re-evaluation of chronological and palaeodietary evidence from human remains. *Journal of Archaeological Science: Reports* 14, 530–542.
172. Piličiauskas, G., Heron, C., 2015. Aquatic radiocarbon reservoir offsets in the southeastern Baltic. *Radiocarbon* 57 (4), 539–556.
