# Supplementary material for: The genetic prehistory of the Baltic Sea region
Source: Nat Commun. 2018 Jan 30;9:442. doi: 10.1038/s41467-018-02825-9 (PMC5789860; doi:10.1038/s41467-018-02825-9)
Supplement: Supplementary file 3 — Description of Additional Supplementary Files [file 41467_2018_2825_MOESM3_ESM.pdf]

Description of Supplementary Files

File Name: Supplementary Data 1
Description: Overview of sample information. Tabular compilation of archaeological information, processing strategy and results for all analysed samples and corresponding experimental blanks.
